# Ethnomedical Knowledge of Plants Used in Traditional Medicine in Mampa Village, Haut-Katanga Province, Democratic Republic of the Congo

**DOI:** 10.1155/tswj/2635735

**Published:** 2025-06-24

**Authors:** Bashige Chiribagula Valentin, Biayi Benaja Martin, Bakari Amuri Salvius, Lumbu Simbi Jean Baptiste

**Affiliations:** ^1^Department of Pharmacology, Laboratory of Therapeutic Chemistry and Analysis of Natural Substances, Faculty of Pharmaceutical Sciences (UNILU), Lubumbashi, Democratic Republic of the Congo; ^2^Department of Pharmacology, Laboratory of Pharmacognosy, Faculty of Pharmaceutical Sciences, University of Lubumbashi (UNILU), Lubumbashi, Democratic Republic of the Congo; ^3^Department of Chemistry, Faculty of Sciences, University of Lubumbashi (UNILU), Lubumbashi, Democratic Republic of the Congo

**Keywords:** alternative medicine, *Anisophyllea pomifera*, *Brachystegia boehmii*, *Entandrophragma delevoyi*, *Landolphia kirkii*, *Pterocarpus brenanii*

## Abstract

The inhabitants of the village of Mampa have developed a rich corpus of knowledge and practices for treating pathologies using plants that are worthy of preservation, perpetuation, and promotion. They draw on the region's rich biodiversity, particularly in the Miombo clear forest. However, to date, no documentation of their ethnomedicinal knowledge exists. This descriptive cross-sectional study was conducted between November 2022 and October 2023. It employed a direct, face-to-face interview with the Mampa village population and a guide questionnaire. A total of 400 respondents were included in the study (sex ratio M/F = 0.9; mean age: 48.0 ± 4.0 years; experience: 14.5 ± 2.0 years), and the majority (93.8%) reported that they learned about plants from their families. These individuals mainly use plants as a first-line treatment (100%) and provided information on 38 plants. The most commonly cited species were *Anisophyllea pomifera* and *Brachystegia boehmii* with 46 citations, while the most commonly used plant was *Landolphia kirkii* with six recorded uses. This is the first report of *Entandrophragma delevoyi* and *Pterocarpus brenanii* as medicinal plants. Most of these plants are trees, comprising 29 from 23 genera belonging to 24 families, with a notable prevalence of Fabaceae (10 plants). Thirty-two diseases are indicated for treatment, with a predominance of gastrointestinal disorders (8 recipes, 7 plants, 152 citations). The root is the most used organ, with 21 recipes and 14 plants, while decoction is the most common preparation method, with 41 recipes and 19 plants. This study's findings indicate that a significant number of medicinal plants are used in traditional Mampa medicine to treat various diseases. Some of these species are endemic to the Miombo biodiversity, while others are shared with other cultures and regions. A series of pharmacological studies are currently underway to validate some of the reported plant indications.

## 1. Introduction

Traditional medicine is the original form of healthcare [1–3]. The plant has been utilized for centuries by many cultures as a primary source of therapeutic agents, frequently constituting a dominant element within traditional medicinal systems [4, 5]. This remains the case despite the evolution of medicine, with more than three-quarters of drugs currently being of biological origin [6, 7]. The advent of technology has resulted in a decline in the prominence of traditional medicine, leading to its marginalization in some regions, and the emergence of alternative or complementary forms of medicine [8]. Unfortunately, the remedies used in biomedicine have not always met expectations. Resistance has been observed in several therapeutic classes of drugs, and the emergence of metabolic pathologies has prompted the search for new remedies [9–11].

Traditional medicine is a highly credible alternative to biomedicine in primary healthcare and the search for new molecules due to its frequency of use, effectiveness, acceptability, and accessibility [12]. Eighty-eight percent of WHO member countries report a high level of use of traditional medicine in their healthcare systems, and in many parts of the world, it remains the only form of treatment available [12]. On the other hand, several molecules currently used in therapeutics are of natural origin and derived from ethnomedicinal knowledge [13, 14]. However, while traditional medicine has been documented and archived in certain civilizations, such as China [15], India [16], and Persia [17], it remains poorly preserved and little disseminated in various African regions, even though, in most cases, it constitutes a form of knowledge accumulated over several generations and has long been an integral part of cultural heritage and local traditions.

In the Democratic Republic of the Congo (DRC), as in many African countries, traditional medicine was the only source of healthcare during the precolonial period [18–21]. Currently, it remains in great demand in several regions of the country, as attested by various ethnobotanical studies carried out in the various areas of DRC, such as Bukavu [22, 23], Uvira [24], Butembo [25], Kisantu and Mbanza-Ngungu [26], Kasangulu [27], Kinshasa [28], Yakoma [29], Kenge [30], Kasai Oriental [31], and particularly Haut-Katanga [32–41].

The results of ethnobotanical studies conducted in Haut-Katanga indicate that 76.4% of the population utilize traditional medicine as a complement to biomedicine [21, 42]. The reasons for turning to traditional medicine in this region are not only accessibility but also the conviction of a certainty of cure [21, 42, 43]. Healing in the region's main urban areas is culturally mixed, with knowledge passed not only within families but also between ethnic groups, where the Bemba, Luba, and Tabwa are dominant [32, 36, 38].

It should be noted, however, that most of these studies have been carried out in large cities, leaving out the villages in between. Given that the transmission of knowledge in the region is from ancestor to descendant and that in Congolese villages, where knowledge is still transmitted orally, with all the risks this entails, it is often people from the same tribe who live there, we thought it would be interesting to integrate this village-based approach into the search for the value of traditional Katangese knowledge used to cure pathologies. With this in mind, we turned our attention to the village of Mampa, located between the city of Lubumbashi and the town of Kasumbalesa, which borders Zambia. This village is so special because traditional medicine is the only form of primary healthcare available, especially since there are no modern health facilities in the village.

This study is aimed at reporting the knowledge, attitudes, and practices of traditional medicine in the village of Mampa, particularly the plants used as medicinal plants.

## 2. Materials and Methods

### 2.1. Experimental Framework

**Figure 1:**
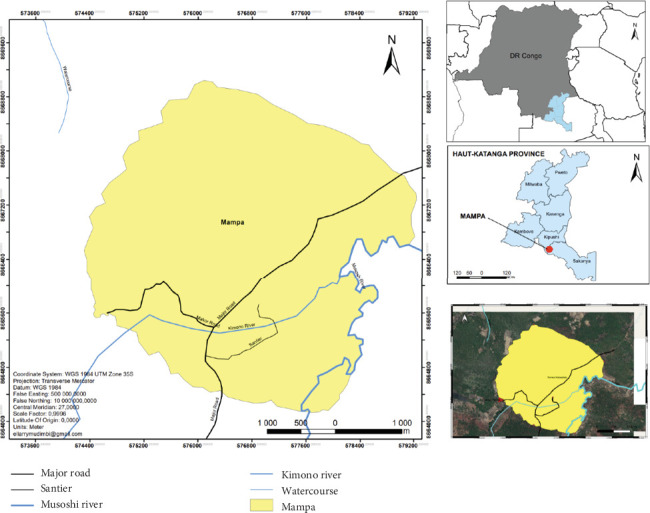
Mampa village, Haut-Katanga, DRC.

The study was conducted in the Mampa village (Figure 1).

The village of Mampa belongs to the Kaponda chiefdom, in the Dilanda groupement, Kipushi territory, Haut-Katanga province, DRC. It was founded in 1925 by Mampa of the Lamba tribe. The village, which lies between 11°40⁣′–12°18⁣′ south latitude and 27°18⁣′–27°45⁣′ east longitude, covers an area of 453.1 km^2^ at an altitude of 1240 m, 10 km from the town of Lubumbashi and 83 km from the city of Kasumbalesa, and is inhabited by the Lamba, Tabwa, Tshokwe, Bemba, Sanga, and Luba-Kas peoples. They form around 600–820 households, with an average of 6 people per household. Their main activities are agriculture and the ember trade. Its geographical location is characterized by a tropical climate, with an average annual temperature of 22.4°C and average yearly rainfall of 512.7 mm^3^. The village has two seasons, with a relatively shorter rainy season from November to April. Characteristic vegetation is of the clear Miombo forest type. The village has no health facilities or schools. All pathologies are treated in the first line of traditional medicine.

### 2.2. Ethnomedical Data Collection

This is a descriptive cross-sectional study. It was conducted among the population living in the village of Mampa between November 2022 and October 2023 in a direct interview using a guide questionnaire. Questions focused on the village population's practices and attitudes to various diseases and the plants used to treat them. In addition, information relating to some of the sociodemographic characteristics of the population surveyed was also collected. The following formula determined the size of the representative sample [40, 44]:

(1) $$\begin{array}{c} n=\frac{p\times \left(1-p\right)\times N\times t_{p}^{2}}{t_{p}^{2}\times p\;\left(1-p\right)+\left(N-1\right)\times y^{2}} \end{array}$$

where *n* is the sample size, *N* is the actual population size (4926), *p* is the expected proportion of respondents (estimated *p* at 0.5), *t*_*p*_ is the sampling confidence interval (*t*_*p*_ = 1.96 given that we set a 95% confidence interval), and *y* is the margin of sampling error: we set this at 0.05. This yields a household population of 356. We went as far as 400 people, further reassuring the representativeness of our sample.

People living in the Mampa village who had already used medicinal plants to treat illnesses were included in the study, if they had been met in the various households of the Mampa village, that they had agreed to take part in the research and that they had been able to answer the questions at the time of the interviews. The interviews were conducted in Swahili, the language spoken in the village by the entire population, and the local ethnic languages.

### 2.3. Data Processing and Analysis

The names of the plant species were given by the respondents in the local language during the interviews. The plants were collected in the company of the interviewees. On this occasion, photographs were taken, and herbarium specimens were prepared and deposited at the Kipopo Herbarium, where the plants were formally identified, and given scientific names by the herbarium botanists. The names were updated by comparison with the following databases: Plants of the World Online (https://powo.science.kew.org/), The World Flora Online (http://www.worldfloraonline), or the African Plant Database (https://africanplantdatabase.ch/). Similarly, the geographical distribution of plants is based on POWO data (https://powo.science.kew.org/). Chi-square tests were used to study correlations between qualitative variables. Where some observed numbers were less than 5, the exact Fischer test was used, since the chi-square test is inapplicable in this case. The significance and confidence levels were fixed, respectively, at 5 and 95%. Statistical processing has been performed with GraphPad Prism 10.4.2.

Given the current controversy surrounding ethnobotanical indexes [45], we have used only the terms numbers, relative frequency citation (RFC), usual value (UV), and percentage to assess the results of our investigations quantitatively.

### 2.4. Ethics Approval, Consent to Participate, Research and Availability of the Study

The Department of Pharmacology, Faculty of Pharmaceutical Sciences, University of Lubumbashi (FSPUNILU-DP-BD-062022), reviewed and approved the project proposal and ethical rules. Before the start of data collection, in accordance with the ethical standards of the University of Lubumbashi, all respondents (*n* = 400) were asked to provide voluntary verbal consent. All participants consented to participate in the study in the presence of a representative of the village head and, in some cases, of the nearest neighbor. Informants were automatically excluded from the study if they did not give their informed verbal consent. All respondents were informed that the data collected would be used for academic purposes only.

The preprint version of this study has been available online on Research Square since the 23rd of September 2024 (https://www.researchsquare.com/article/rs-5116022/v1).

## 3. Results and Discussion

### 3.1. Knowledge, Attitudes, and Practices of the People of Mampa Village Who Use Plants to Treat Various Pathologies

There are six main reasons why people in the village of Mampa turn to medicinal plants, with the most cited reasons being family tradition (93.8%), distance from hospitals (25.3%), and certainty of cure (23.8%). Mampa people turn to six primary sources for their herbal remedies, dominated by friends (24.5%) and family members (24%). But long before resorting to herbal remedies, the Mampa people, depending on their pathologies, resort to five practices, of which the analysis of signs, symptoms (35%), and tests derived from the ancestral tradition (24%) are reported by nearly three-fifths of those surveyed (Table 1).

Although most of the people in the village of Mampa use plants to treat their illnesses and, in most cases (49%), they are not aware of the risks involved, some of them are aware of certain risks associated with the use of medicinal plants. Of the four risks mentioned, not completing the cure (13%) or aggravating the illness (11.5%) are the two most frequently cited reasons. The Mampa population is aware that the use of medicinal plants does not always produce the expected results and that there have been cases of therapeutic failure. When such cases occur, seven attitudes are observed by the Mampa villagers, of which the two most frequently mentioned are either to do nothing (24%) or to change the plant (22%) (Table 1).

### 3.2. Knowledge of Medicinal Plants Among the Population of Mampa Village

In total, 38 plants are used by the population of Mampa village to treat 33 pathologies, with *Anisophyllea pomifera* (46 citations) being the most cited plant and *Brachystegia boehmii* (5 uses) being the most used plant. The characteristics of these plant relate to their family, morphological, and geographical types, as well as the characteristics of the medicinal recipes derived from their use (Table 2).

### 3.3. Morphological and Geographical Type

**Figure 2:**
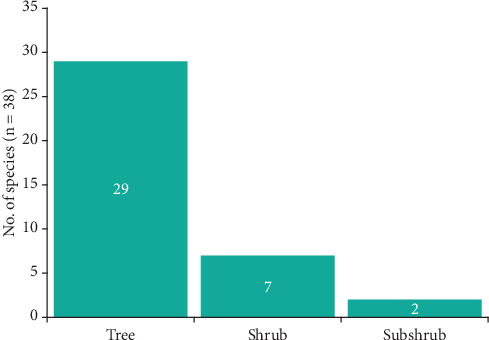
Morphological types of medicinal plants from Mampa village.

**Figure 3:**
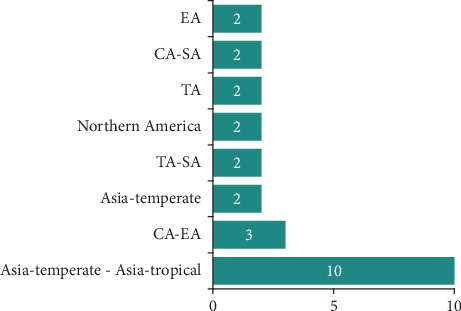
Geographical types of medicinal plants in Mampa village (type with more than one taxon). *Legend*: CA, Central Africa; SA, Southern Africa; MA, Madagascar; EA, East Africa; NA, North Africa; WA, West Africa.

The medicinal plants inventoried in the village of Mampa can be classified into three morphological types, with the tree type representing 76.3% of the total (Figure 2). They can be further divided into 22 geographical types, with plants endemic to Asia representing the majority. However, African endemic plants account for 47.4% of all plants, including 13 from the DRC (Figure 3).

### 3.4. Family and Local Name

**Figure 4:**
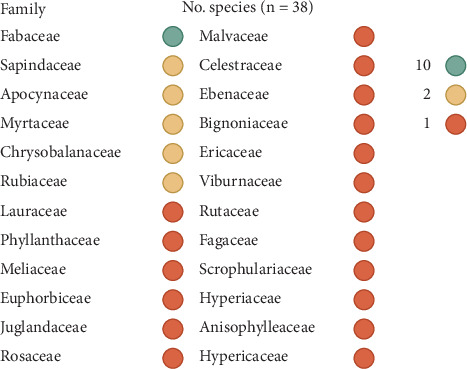
Botanical families of medicinal plants from the Mampa village (*n* = 38). *Legends*: The intensity of the color corresponds to the number of plants in the family concerned, according to the scale shown in the figure.

**Figure 5:**
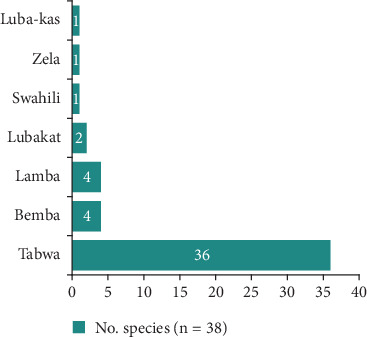
Local languages used to name medicinal plants in Mampa village.

The 38 plants are distributed among 23 genera belonging to 24 families. The most prevalent family is Fabaceae, which accounts for 26.3% of the plants, followed by four families with two plants each: the remaining families are represented by only one plant each (Figure 4). These plants are named in seven local languages of the DRC, of which Tabwa is the most represented with 36 plants. It is followed by Bemba and Lamba with four plants each (Figure 5).

### 3.5. Used Part and Mode of Preparation

**Figure 6:**
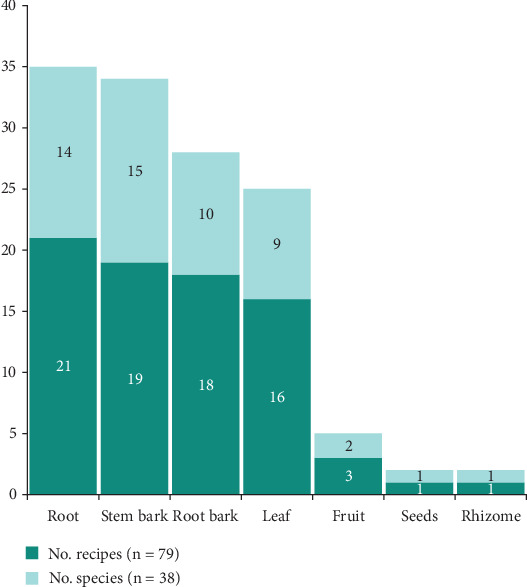
Parts of the medicinal plants from Mampa village.

A total of 79 single plant medicinal recipes and two mixed plant medicinal recipes were derived from the 38 plants using seven plant parts. The root was the most utilized plant part, with 21 recipes and 14 plants, followed by the stem bark, which was used in 19 recipes and with 15 plants. The root bark was the third most utilized plant part, with 18 recipes and 10 plants (Figure 6).

**Figure 7:**
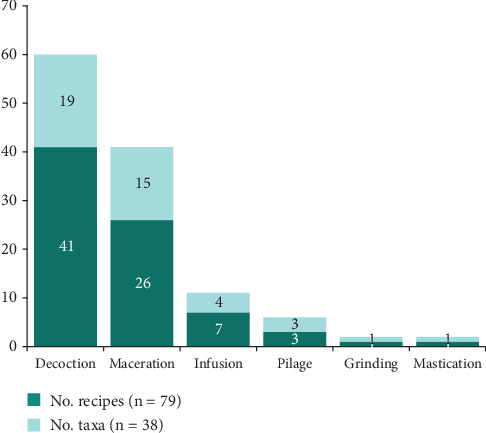
Methods of preparing herbal remedies in the village of Mampa.

The seven organs utilized in a multitude of medicinal preparations are processed via six distinct methods, with decoction being the predominant approach, accounting for 41 recipes and 19 plants (Figure 7). This is followed by maceration, which is employed in 26 recipes and 15 plants.

### 3.6. Ailments Treated by Plants Inventoried in Our Experimental Framework

**Figure 8:**
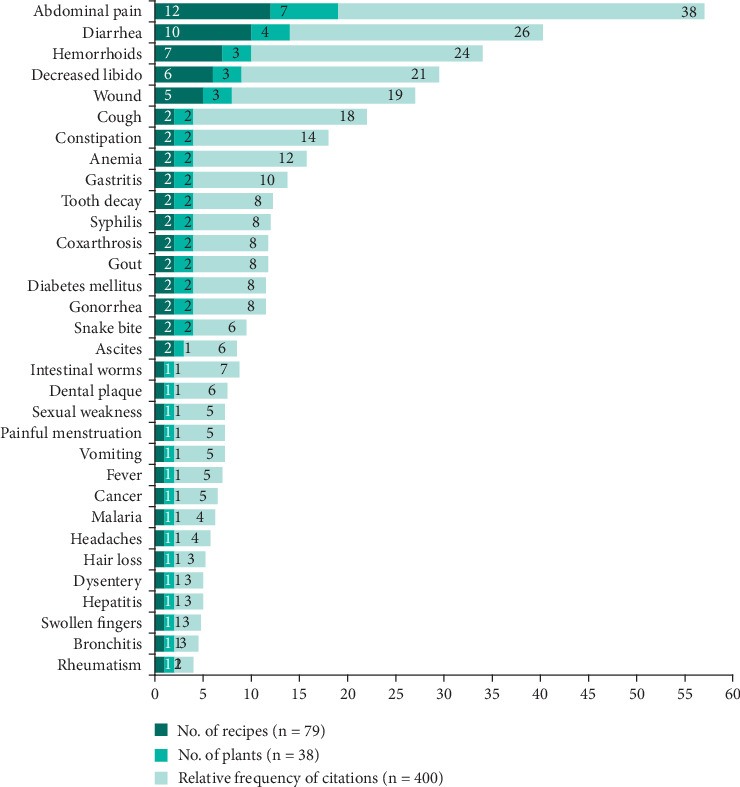
Signs and ailments treated by traditional medicine in the Mampa village.

The 79 recipes from 38 plants inventoried in this study are used in the treatment of 32 ailments where abdominal pain (12 recipes, 7 plants, 152 citations), diarrhea (7 recipes, 4 plants, 104 citations), hemorrhoids (4 recipes, 3 plants, 96 citations), decreased libido (3 recipes, 3 plants, 76 citations), and cough (3 recipes, 2 plants, 72 citations) are treated by at least 3 medicinal recipes (Figure 8).

### 3.7. Previous Medicinal Knowledge of the Organs of Inventoried Plants

A review of the existing literature (Table 3), focusing on the last medicinal uses of the 38 plants used in the Mampa village, reveals the following:

i. Eight plants had previously been reported as medicinal plants in Katanga, of which five (*Anisophyllea boehmii*, *Brachystegia boehmii*, *Crossopteryx febrifuga*, *Harungana madagascariensis*, and *Landolphia kirkii*) have the same medicinal uses in Mampa as in the existing literature from the region and three (*Parinari curatellifolia*, *Pterocarpus angolensis*, and *Senegalia polyacantha*) have new medicinal uses.
ii. Three plants are reported as medicinal plants in regions of the DRC other than Katanga and for ailments other than those for which they are used in the village of Mampa: *Kigelia africana*, *Ricinus communis*, and *Syzygium cumini*.
iii. Nine plants have the same medicinal use in Mampa as in other regions outside the DRC: *Albizia julibrissin*, *Bauhinia variegata*, *Buddleja davidii*, *Cupaniopsis anacardioides*, *Damnacanthus indicus*, *Juglans nigra*, *Phoebe lanceolata*, *Piliostigma reticulatum*, and *Rhododendron simsii*.
iv. Two plants, *Entandrophragma delevoyi* and *Pterocarpus brenanii*, are reported for the first time as medicinal plants. Both are endemic to Africa, one of which, *E. delevoyi*, is singularly endemic to the DRC. These plants constitute the particularity of the Mampa village from the point of view of ethnomedicinal uses of plants.
v. Sixteen plants are reported as medicinal plants in other regions outside the DRC and for uses other than those for which they are used in the village of Mampa: *Butea monosperma*, *Chrysobalanus icaco*, *Cratoxylum cochinchinense*, *Dimocarpus longan*, *Diospyros virginiana*, *Dombeya rotundifolia*, *Elaeodendron orientale*, *Flueggea suffruticosa*, *Gomphocarpus sinaicus*, *Piscidia piscipula*, *Prunus armeniaca*, *Psidium cattleianum*, *Pueraria montana*, *Quercus suber*, *Tetradium ruticarpum*, and *Viburnum odoratissimum* (Table 3).

### 3.8. Important Medicinal Plants in Mampa

In this study, we consider important local medicinal plants to be those that meet one of three criteria: (i) plants with the highest number of citations, (ii) plants with the highest number of uses, and (iii) plants that have been identified as medicinal for the first time.

**Figure 9:**
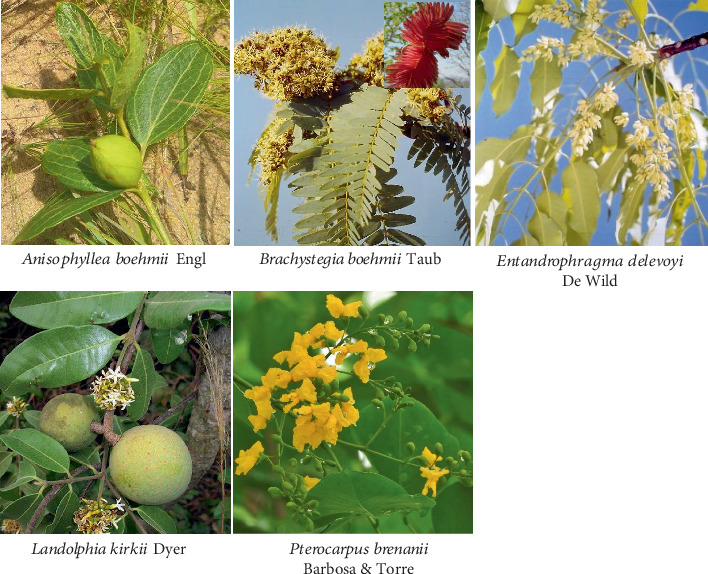
Plants particularly highlighted by the study.

The most frequently cited plants are *Anisophyllea pomifera* and *Brachystegia boehmii*, with 30 citations each. In contrast, the most frequently utilized plants are *Landolphia kirkii* (six uses) and *Brachystegia boehmii* (five uses). This study marks the first time *E. delevoyi* and *Pterocarpus brenanii* have been identified as medicinal plants (Figure 9).

### 3.9. The Sociodemographic Characteristics of the Interviewees

Four hundred resource persons were interviewed as part of this study, with an average age of 48 ± 4 years (ranging from 24 to 72 years). The male-to-female ratio was 0.99. The respondents were either household representatives (93.75%), herbalists (2.50%), or traditional healers (3.75%). The respondents' mean experience in using medicinal plants was found to be 9 ± 2 years (range: 2–31 years). The participants were engaged in seven primary occupations, with agriculture and livestock breeding representing over 60% of the total. In over 50% of cases, the respondents had only completed elementary school (Table 4).

## 4. Discussion

### 4.1. Knowledge, Attitudes, and Practices of Traditional Medicine Using Plants in the Mampa Village

The interviews conducted as part of this study revealed that all subjects consulted utilized traditional medicine, predominantly based on plant use. This result is consistent with the findings of a recent study conducted in Lubumbashi, which revealed that 76.4% of the population utilizes traditional medicine, with a notable prevalence of plant-based remedies [21]. This observation has been previously documented in ethnobotanical studies conducted in Haut-Katanga, although these studies were oriented toward specific pathologies [32–34, 35, 36–38, 40, 41].

This study identified family tradition as the primary rationale for the utilization of plants in traditional medicine, a finding that aligns with those of previous research studies [99–102]. The impact of family tradition on the uptake of traditional medicine is a complex phenomenon. These include the following: (i) The practice may be regarded as a cultural heritage that has become an integral part of the family's identity and heritage [103]. An alternative perspective is that it may be viewed as a means of symbolizing continuity, respect for ancestors, and the link with cultural roots, which may serve to reinforce the feeling of belonging to a community [104]. Thirdly, some families may perceive this practice as a means of achieving greater autonomy and control over their well-being [105]. Fourthly, the practice may have given rise to a positive family tradition passed down through generations. Alternatively, it may serve as an element of family cohesion and reinforcement of the unity of a community, as observed by parents and relatives. In this context, the certainty of cure, identified by this study as a reason for the use of traditional medicine by nearly 24% of the population surveyed (Table 1), may appear to be a secondary justification for the use of traditional medicine based on family practice.

The second most frequently cited rationale for using traditional medicine in this study was the distance to healthcare facilities (Table 1). The lack of physical accessibility to biomedicine has been previously identified as a rationale for using traditional medicine. This is particularly evident in studies conducted in various African countries, including Uganda [106], Rwanda [107], Ethiopia [108, 109], Kenya [101], Nigeria [110], Cameroon [111, 112], Zambia [113], and the DRC, particularly in Haut-Katanga [21]. Although poor accessibility is a common and widely reported reason for the use of traditional medicine in many regions, in the case of the village of Mampa, traditional medicine is the only alternative, given that the village lacks a health structure where modern medicine is practiced. This situation serves to illustrate the role of traditional medicine in primary healthcare in the village of Mampa. These considerations can also be correlated with the sources of knowledge evoked by the population interviewed on the use of traditional medicine, where friends, family, and previous experience figure prominently (Table 1).

It is evident that the diagnostic procedures employed in traditional medicine in the Mampa village are not distinctive when compared to the methods of diagnosis documented in the literature on the diagnosis of pathologies in African traditional medicine [114, 115]. This is particularly the case in the context of the Katanga region [38, 43]. Notably, clinical signs represent the primary foundation for diagnosis in traditional medicine within the Mampa village (91%). The utilization of clinical signs for the diagnosis of illnesses in traditional medicine offers several advantages, including (i) the establishment of a link between traditional medicine and biomedicine, the latter also employing clinical signs to connect manifestations to specific drugs, and (ii) the facilitation of the development of an experimental procedure, particularly in clinical studies, aimed at validating traditional medicine practices [116]. However, this approach has the disadvantage of exposing the patient to the risk of incomplete treatment, of which only a minority of the population surveyed (13%) was aware (Table 1).

Regarding the knowledge of the risks associated with the use of plants in traditional medicine in the village of Mampa, it is notable that several risks identified in this study have been previously documented in other studies [117, 118]. However, some risks have not been previously reported. These include the risk of plant confusion during harvesting, intoxication by the metallic trace elements (TMEs) that specific plant concentrate, and the ecological risk. It is particularly noteworthy that nearly half of the population is unaware of the potential risks associated with using medicinal plants. This could have a detrimental impact on the social stability of the village, particularly given that therapeutic failures may be attributed to witchcraft or mystical practices.

In the event of therapeutic failure when using medicinal plants, two attitudes are predominant in the Mampa village: inaction (24%) and changing the plant (22%). Those who elect to do nothing in the event of therapeutic failure adhere to the belief that there is no alternative to traditional medicine, as they perceive this approach to be infallible. This viewpoint has also been documented in prior research conducted in the region [21, 43]. This indicates the degree of belief in traditional medicine held by this segment of the population, who have likely never encountered a genuine case of therapeutic failure. The remaining majority opinion is to change the plant. This approach is analogous to that of biomedicine, wherein a change in medication is observed in the event of therapeutic failure. This suggests a spectrum of treatment options within traditional medicine in Mampa. Consequently, the mention of two plants in the management of pathology does not necessarily imply that they are used alternatively but that they may also be employed as a palliative measure. It is important to consider this information when collecting ethnobotanical data on traditional Katangan medicine.

### 4.2. A Variety of Medicinal Plants From the Mampa Village

Traditional medicine in the village of Mampa uses 38 plants. Most of these plants are endemic Asian trees belonging to the Fabaceae family, most of which are named in Tabwa (Table 2).

As evidenced by various ethnobotanical studies conducted in the region [33, 34, 38, 43, 119], the predominance of trees among medicinal plants in Haut-Katanga is a well-documented phenomenon. In contrast to other morphological types, using trees for therapeutic purposes offers several advantages. First, the availability of raw materials is not limited by seasonality, allowing for year-round utilization. Second, using different organs can increase the versatility of the therapeutic supply. Third, the low probability of extinction of highly utilized plants is another advantage.

Many plants inventoried in this study originate from Asia, but only three plants are endemic to the DRC. This differs from various ethnobotanical studies conducted in the Katangese region [36, 39, 120, 121]. The high prevalence of nonendemic medicinal plants in the village of Mampa may indicate a culturally influenced tradition of traditional medicine in this village, potentially shaped by Asian cultural influences. The presence of an Indian agricultural enterprise in the region for nearly two decades supports this hypothesis. However, this hypothesis may be called into question not only by the fact that all the medicinal plants listed are named in local languages, with a predominance of Tabwa, an ethnic group reputed to be among the healing peoples of Katanga, but also by the fact that the acquisition of medicinal knowledge in this village is, in half the cases, the result of a family tradition, dating back to the creation of the village, according to the information gathered during our interviews (Table 1). In the latter case, the plants in question would not have been formally identified as endemic to Katanga.

The preponderance of Fabaceae among the plants inventoried during this study agrees not only with previous ethnomedicinal studies carried out in the area covered by the Miombo open forest [56, 122–126] but also with ethnomedicinal studies carried out in Katanga [36, 38, 39, 43, 127]. This numerical predominance of Fabaceae is globally observed across all plants in sub-Saharan Africa and has been attributed to their ability to capture atmospheric nitrogen, enabling them to grow in any soil type, whether nutrient-rich or nutrient-poor [28, 128].

In the Mampa village, the majority of plants used for therapeutic purposes (94.7%) are wild species. This finding is consistent with the results of previous ethnobotanical studies conducted in the same region [35, 36, 38–40, 127, 129]. The predominant reliance on wild plants as medicinal resources presents both advantages and risks, necessitating a balanced approach. The availability of wild plants without the need for intensive cultivation minimizes ecological impact by reducing pesticide use and preserving local biodiversity. Furthermore, their traditional medicinal applications are often deeply rooted in local knowledge, making them accessible to rural communities, particularly in areas with limited medical infrastructure, such as Mampa village. However, excessive dependence on these uncultivated species poses several risks. The overexploitation of natural resources can have severe consequences, including a decline in population species numbers, which in turn can hinder the ability of these populations to regenerate. This decline can even lead to the extinction of some species. The biochemical composition of wild plants varies significantly due to environmental factors, which complicates the standardization of treatments and raises concerns regarding safety and therapeutic efficacy. Additionally, their occurrence in remote locations may limit access, exacerbating disparities in healthcare availability. Lastly, the absence of environmental control can expose these plants to contaminants such as pollutants and toxins, which may alter their medicinal properties. Therefore, while wild plants play a crucial role in traditional pharmacopeia, their sustainable utilization requires a carefully managed approach that ideally integrates cultivated species to ensure long-term preservation and accessibility.

### 4.3. Pathologies, Diseases, Signs, or Conditions Treated by Medicinal Plants in the Mampa Village

The results of our surveys indicate that four ailments or conditions are the most prevalent in the Mampa village, with a total of 32 identified. These are gastrointestinal disorders, diarrhea, hemorrhoids, and low libido. Fortunately, the local traditional medicine has plants that can treat these conditions, with seven plants for gastrointestinal disorders, four plants for diarrhea, and three plants for libido and hemorrhoids, respectively (Figure 8).

At the national level, the 10 most prevalent and lethal pathologies in the DRC are malaria, tuberculosis, lower respiratory tract infections, neonatal diseases, diarrheal diseases, strokes, ischemic heart disease, road trauma, hypertensive heart disease, and cirrhosis and other chronic liver diseases [130]. The findings of our surveys indicate that the medicinal knowledge of the Mampa village population may be associated with three of the 10 leading causes of mortality: diarrhea (four plants), malaria (one plant), and lower respiratory tract infections (two plants). These two scenarios, at the local and national levels, illustrate the efficacy of traditional medicine in addressing significant health concerns. Furthermore, these findings underscore the significance of traditional medicine in rural communities, particularly in the context of primary healthcare, as evidenced by numerous prior studies [131–133].

The village of Mampa has been documented to have a variety of ethnomedicinal uses, which have also been observed in other regions of the country and the world.

The ethnobotanical uses reported later in Katanga (DRC) are also observed in the Mampa village. These include the decoction of *Anisophyllea pomifera* Engl. & Brehmer roots, used in Kipushi (Haut-Katanga, DRC) to treat diabetes [36], and that of *Harungana madagascariensis* root bark, used in the Kafubu valley (Haut-Katanga) against rheumatism [36, 41]. These same uses are reported in the Mampa village. Similarly, the leaves of *Landolphia kirkii* are employed in Lubumbashi as a decoction to treat malaria [34, 37]. Additionally, the stem barks of *Parinari curatellifolia* are utilized in Lubumbashi as a decoction for managing diarrhea [37]. *Pterocarpus angolensis* stem bark is employed in Lubumbashi as a decoction for treating diabetes [34, 36, 37]. Ultimately, *Senegalia polyacantha* stem bark is employed in Lubumbashi as a decoction or infusion for managing diarrhea and diabetes, respectively [34, 36]. The uses above were similarly documented in the Mampa village as part of the present study.

Other medicinal applications, documented in both Katanga and other regions, were observed in the Mampa village during our research. These include *Crossopteryx febrifuga* stem bark, which has been used as a decoction to treat malaria and diarrhea in Kipushi in the DRC [36, 38, 41] and in other countries such as Guinea, Nigeria, and Zimbabwe [64]. Similarly, *Brachystegia boehmii* leaves are employed in the form of a compress in the Likasi region of Katanga, DRC [39], and as a decoction in Zimbabwe [55] for the treatment of wounds. Furthermore, its ashes are utilized as a therapeutic agent against gonorrhea in the regions as mentioned above [54].

Other ethnopharmacological applications of Mampa have been documented in other global cultures situated outside the DRC. For example, *Buddleja davidii* roots are used in China against wounds [58] and the Republic of Korea [59]. *Butea monosperma* roots are used in India against snake venom [61]. In China, *Damnacanthus indicus* roots are used against cancerous pathologies [66]. The root barks of *Juglans nigra* are employed in the United States for the management of diarrheal pathologies [74]. The roots of *Phoebe lanceolata* are utilized in China to manage wounds [77]. The stem bark of *Piliostigma reticulatum* is used in Senegal [79] and Ivory Coast [78] for the treatment of diarrheal disorders. Based on the findings of our investigation, which are presented in this study, the same ethnopharmacological uses are reported for the same plant in the Mampa village.

The above-cited consensuses, obtained from individuals of disparate backgrounds, demonstrate that there is reason to accord some degree of credibility to the findings of our investigation. Furthermore, they suggest that the results of prospective biological experiments conducted in a laboratory setting are likely to corroborate these uses [38, 39].

### 4.4. Medicinal Recipes From Mampa Village

The population has identified 32 pathologies in the Mampa villages and developed 81 recipes to treat these conditions, of which two employ two plants and 79 utilize a single plant per recipe. The root is used in 21 recipes and 18 recipes for root bark (see Figure 6), while decoction (see Figure 7) is the most commonly utilized preparation method and plant part.

The preponderance of roots as the part used is also reported in some targeted ethnomedicinal studies previously carried out in the region [32, 34, 39, 41, 119, 134]. In contrast, other targeted ethnomedicinal studies have indicated that the leaf is the most commonly used part [33, 35, 37, 40]. The lack of consensus on which organ is the most widely used in traditional medicine in Katanga may be justified by the diversity of localization of bioactive phytochemical compounds within the plant, which can vary from one pathology to another. The utilization of roots offers certain advantages, including providing primary metabolites, vitamins, and minerals such as carbohydrates, pectin, calcium, and vitamin A [135]. Furthermore, the roots can provide access to secondary metabolite groups, including anthocyanins [136], flavanones and flavanols, lipids [136], and phenylpropanoids and organo-heterocycles [127]. However, reliance on the root exposes the plant to extinction, as it is in high demand. It would, therefore, be advantageous for the residents of Mampa to receive instruction in techniques that facilitate the natural regeneration of plant material, which is frequently utilized for therapeutic purposes.

In local traditional medicine, decoction is the preferred method of preparation, as evidenced by the findings of several studies conducted in Haut-Katanga [32–34, 35, 36–41, 119, 134].

In the context of traditional medicine, the preparation of a medicinal formula via decoction offers several advantages. Firstly, this preparation method is optimal for extracting active compounds that remain stable at high temperatures. Secondly, the process does not require the use of sophisticated or costly apparatus, thereby facilitating its implementation on a vast scale. Thirdly, the method is relatively straightforward and does not necessitate the involvement of a skilled operator, which is advantageous in traditional settings. Fourth, it is a cost-effective method that can be readily implemented in a domestic setting. However, decoctions are not an optimal method for the extraction of heat-sensitive components, as the boiling process can lead to their degradation [40, 137–139]. Nevertheless, decoction should not be the default preparation method in a specific local culture. The optimal preparation method should be determined on an individual basis, considering the specific pathology and the nature of the remedy. Only through rigorous biological experimentation can the rational use of each preparation method be accurately assessed.

### 4.5. Essential Medicinal Plants Discovered in the Mampa Village

The most frequently cited plants in this study are *Anisophyllea pomifera* and *Brachystegia boehmii*, while the most widely used plant is *Landolphia kirkii*. *E. delevoyi* and *Pterocarpus brenanii* represent the plants first reported as medicinal plants. It is thus possible to consider these plants as those which have been singled out by this study in the context of Mampa village.

*Anisophyllea pomifera* is a tree species endemic to the southwestern part of Africa, including countries such as Tanzania, Malawi, Zambia, Burundi, and the DRC (https://www.worldfloraonline.org/taxon/wfo-0000537325). In Tanzania, *Anisophyllea pomifera* is utilized as a means of deterring rodent pests that prey on maize and beans, and its fruit is consumed [140, 141], as is the case in Burundi [140]. In the DRC, in the southern Katanga region, the decoction of *Anisophyllea pomifera*'s leaves, roots, and stem bark is utilized for the treatment of dental caries, malaria, abdominal discomfort, mental health disorders, hypertension, and respiratory conditions, including coughs [34, 35]. Some of these applications, in addition to indications against urinary tract infections and diabetes, were documented in the Mampa village during our various surveys (Table 2). In vitro studies have demonstrated that the plant's methanolic leaf extract exhibits antimicrobial activity against *Streptococcus mutans* MTCC 890 (minimum inhibitory concentration (MIC): 31.25 *μ*g/mL) and *Lactobacillus acidophilus* MTCC 447 (MIC: 62.5 *μ*g/mL) [35]. Additionally, the methanolic root extract demonstrated in vitro antiplasmodial activity against *Plasmodium falciparum*, with an IC_50_ of 22.1 *μ*g/mL [50]. Additionally, the root was found to contain saponins, tannins, and terpenoids, while the leaves were also found to contain alkaloids and steroids. However, this plant has the same vernacular name (Fungo in Sanga and Lufunga in Tabwa) as *Anisophyllea boehmii* Engl., with which it shares certain medicinal uses, notably in the management of diabetes and digestive disorders in Haut-Katanga [36]. This suggests a potential case of confusion in the use of these two plants in traditional Katangese medicine. Subsequent simultaneous pharmacognostic and phytochemical studies on both plants would be beneficial to reach a definitive conclusion.

*Brachystegia boehmii* Taub is an endemic African tree species that is native to the following countries: The species is found in Angola, Botswana, Burundi, Malawi, Mozambique, the DRC, Tanzania, Zambia, and Zimbabwe (https://powo.science.kew.org). The ethnobotanical uses of this plant vary depending on the country and the organ in question. In Mozambique, the roots are employed in a decoction to assuage agitation in patients [142], whereas the leaves are utilized in a decoction to alleviate abdominal discomfort and the effects of snake venom [143]. In Tanzania, the roots are used in a decoction to treat impotence and wounds [54]. In Zimbabwe, the leaves are employed in a decoction for the treatment of wounds, constipation, lumbago, back pain, and dysmenorrhea [55, 142]. Additionally, macerated stem bark is indicated for the management of sexually transmitted infections [144]. In the DRC, the roots are employed as a macerate to treat wounds and coughs, whereas the stem barks are utilized as a decoction to address dysentery, diarrhea, abdominal discomfort, and gonorrhea [40]. This plant is used globally for the same purposes in the Mampa village (Table 2).

To our knowledge, the only pharmacological evidence reported to date concerns the leaves. Chloroformic leaf extract showed in vitro antibacterial activity against *Pseudomonas aeruginosa* ATCC 27853, *Staphylococcus aureus* ATCC 25923, *Staphylococcus epidermidis*, and *Escherichia coli*, with a DZI ranging from 12 to 21 mm [55]. The ethanolic extract of the leaves showed only weak anti-inflammatory activity of 48% on COX-1 and antioxidant activity with an IC_50_ of 46 *μ*g/mL [145]. The primary metabolite metabolomic profile of fresh leaves provides information on the presence of 39 primary metabolites, including seven amino acids (glycine (Gly), alanine (Ala), valine (Val), glutamate (Glu), *γ*-amino butyric acid (GABA), isoleucine (Ile), and threonine (Thr)), two organic acids (threonate and glycerate), and two sugars (galactose and glycerol) [146].

*Landolphia kirkii* Dyer ex Hook.f is a tree species endemic to southern and central Africa, occurring in the following countries: The species is found in the following countries: Burundi, Central African Republic, DRC, Kenya, Malawi, Mozambique, South Africa, Somalia, Tanzania, Uganda, Zambia, and Zimbabwe. In some regions, various parts of the plant are employed in multiple applications. The fruit is utilized primarily as food in all countries where the plant is endemic, except the DRC, where its decoction is employed for the treatment of constipation [32] and malaria [147]. In South Africa, the roots are employed as an infusion to alleviate dental discomfort [148], diarrhea, and blood purification [149]. In Kenya [150, 151] and Mozambique [56], the plant is used as a decoction to relieve digestive disorders. In the DRC, the plant is used as an infusion to combat urogenital schistosomiasis and intestinal worms [32]. The leaves are primarily utilized in the DRC as a decoction to treat convulsions [50] and as a maceration to combat malaria [50, 152]. The antimalarial use of the plant, as documented in the village of Mampa, is similarly observed in other regions of the DRC [152] and other parts of the continent [153].

In a previous study, the methanolic extract of the leaves demonstrated antiplasmodial activity against local isolates of *Plasmodium falciparum* with an IC_50_ of 9.9 *μ*g/mL [50]. To the best of our knowledge, this represents the only validated in vitro use of the leaves to date. No biological studies have been conducted on the roots and stems. However, the methanolic fruit extract is active in vitro against *Salmonella typhimurium* ATCC 14028, *Streptococcus pyogenes* ATCC 21059, and *Klebsiella pneumoniae* ATCC 13883 with MICs ranging from 6.3 to 24 *μ*g/mL. Nevertheless, it exhibits weak antioxidant activity, with an IC_50_ value greater than 100 *μ*g/mL [154]. A previous phytochemical screening of the fruit revealed the following characteristics of the plant: The chemical composition of the plant includes hexadecanoic acid, 9-octadecanoic acid, tetradecanoic acid, 9,12-octadecanoic acid, and 9,12,15-octadecatrienoic acid [155]. The ash content is reported as follows: The mean value was 2.9 ± 0.0, and the protein content was 12.7 ± 0.3 mg/kg. The results indicated that the sample contained 2.1% ± 0.20% moisture, 0.9% ± 0.1% ash, and 1.8% ± 0.2% fat. The total phenolic content (TPC) was found to be 1250.33 mg of gallic acid equivalent (GAE) per gram of mollified fruit, while the total flavonoid content (TFC) was determined to be 2019.0 mg of rutin equivalent (RE) per gram of mollified fruit [156].

*E. delevoyi* De Wild is a tree species indigenous to the DRC, Rwanda, and Burundi. To the best of our knowledge, the only data available in the literature concern phytochemistry. These findings indicate that the hexanic extract of the bark of this plant contains azadirone, 14*β*,15*β*-epoxyazadirone, 6*α*-acetoxyazadirone, 6*α*-acetoxy-14*β*,15*β*-epoxyazadirone, and 6*α*-acetoxy-14*β*,15*β*-epoxyazadiradione. Additionally, the phytochemical profile of the bark includes 4-secotirucalla-4(28),7,24-triene-3,21-dioic acid, delevoyin A (3,4-secotirucalla-4(28),7,24-trien-3-oic acid), delevoyin B (6*α*-acetoxy kihada lactone), limonoid, 11*β*-acetoxygedunin, and delevoyin C [157]. In the village of Mampa, *Entandrophragma delevoyi* is employed to treat malaria. Despite the absence of prior documentation regarding this utilization in other regions, a range of *Entandrophragma* plants are used in traditional medicine as a malaria treatment. These include *Entandrophragma angolense*, *Entandrophragma candollei*, *Entandrophragma caudatum*, *Entandrophragma congolense*, *Entandrophragma palustre*, and *Entandrophragma utile* [158]. The findings of this study thus allow for the inclusion of *E. delevoyi* among the various plants of this genus.

*Pterocarpus brenanii* Barbosa & Torre is a tree species endemic to Malawi, Mozambique, Zambia, and Zimbabwe. Despite a lack of documentation attesting to its presence in the DRC, the proximity of the village of Mampa to Zambia may provide a rationale for its inclusion in the village's traditional medicinal practices. Furthermore, despite the absence of ethnobotanical studies on this topic in the accessible literature, two medicinal uses in the village of Mampa (malaria and cancer) have been documented in various plant of the *Pterocarpus* genus [159, 160]. It would be beneficial to conduct further research to determine whether these two pathologies may serve as pharmacotaxonomic markers for the entire *Pterocarpus* genus.

The two plants, *Pterocarpus brenanii* and *E. delevoyi*, are particularly interesting candidates for further ethnopharmacological studies so that their medicinal uses in the Mampa village can be validated. The same can be said of plants *Parinari curatellifolia, Pterocarpus angolensis*, and *Senegalia polyacantha*, which seem to have some uses specific to the Mampa village.

### 4.6. Limitations of the Study

Despite the methodological efforts to address the significant concerns that arise in ethnomedicinal studies, particularly data reliability, community access, plant identification, and logistical challenges, this study does not claim to have exhausted all knowledge related to the interaction between plants and the Mampa village population. The study does not give the cutus on the use of these medicinal plants. The study was limited to documenting their medicinal use, thus paving the way for ethnopharmacological studies that should prove their efficacy and safety, thus validating these uses. At a later stage, it will be necessary to complete our knowledge of the other uses of these plants by these peoples (cultural, artistic, nutritional, etc.). Subsequent studies will also have to examine the therapeutic peculiarities of each family, especially since, in the Mampa village, knowledge is acquired and transmitted within the family. Moreover, given that therapeutic expertise in the Mampa village is transmitted orally, there is a risk of variability related to the memory and will of the informants. The influence of the use of plants in the treatment of diseases can cause damage to biodiversity. Since this study did not address this aspect, future studies should focus on it.

## 5. Conclusion

This study illustrates that the Mampa village is inhabited by a diverse range of plant species, which are vital for maintaining the village's health system. These plants represent the sole alternative for primary treatment of the various pathologies encountered by the local population. This ethnomedical knowledge is acquired and practiced within the family unit. Some of the plants employed as remedies in this village are shared with other African cultures, while others appear to be exclusive to the region. This new knowledge thus significantly contributes to the existing body of traditional therapeutic knowledge in Katanga, particularly in the Congo more widely. These findings highlight the necessity for developing techniques to preserve the integrity of the Miombo forest, in which the Mampa village is located, and to educate local populations in this regard. Furthermore, the value of indigenous users' knowledge of phytotherapy merits further investigation through phytochemical and biological studies, with the objective of developing phytomedicines.

## Figures and Tables

**Table 1:** Knowledge, attitudes, and practices of traditional medicine in the Mampa village.

**Factor**	**Men (** **n** = 199**)**		**Women (** **n** = 201**)**		**Total (** **n** = 400**)**		**χ** ^2^ ** test**
	**Nu**	**%**	**Nu**	**%**	**Nu**	**%**	
*Reasons why people use medicinal plants*							
Family traditions	179	89.9	196	97.5	375	93.8	*p* = 0.9324
Reliable hospitals far from home	50	25.1	51	25.4	101	25.3	*r* ^2^ = 0.00075
Certainty of healing	47	23.6	48	23.9	95	23.8	
Short treatment times	29	14.6	29	14.4	58	14.5	
Low cost of treatment	27	13.6	27	13.4	54	13.5	
Easy access to plants	24	12.1	24	11.9	48	12.0	
*Sources of knowledge about medicinal plants*							
Friends	48	24.1	50	24.9	98	24.5	*p* = 0.9627
Family	48	24.1	48	23.9	96	24.0	*r* ^2^ = 0.00023
Previous experiences	29	14.6	29	14.4	58	14.5	
Dream	28	14.1	28	13.9	56	14.0	
Traditional healers	24	12.1	24	11.9	48	12.0	
Herbalists	22	11.1	22	10.9	44	11.0	
*Diagnosis of diseases before using plants*							
Signs and symptoms	170	85.2	194	96.5	364	91.0	*p* = 0.9063
Traditional tests	48	24.1	48	23.9	96	24.0	*r* ^2^ = 0.00184
Dream	29	14.6	31	15.4	60	15.0	
Biomedical examinations	27	13.6	29	14.4	56	14.0	
Ancestral rituals	25	12.6	23	11.4	48	12.0	
*Knowledge of the risks of using medicinal plants*							
No	96	48.2	100	49.8	196	49.0	*p* = 0.9860
Failure to complete the course of treatment leads to relapse	27	13.6	25	12.4	52	13.0	*r* ^2^ = 0.00003
Failure to control the dose	24	12.1	22	10.9	46	11.5	
Worsening of the disease	20	10.1	22	10.9	42	10.5	
Initiation to mystical practices	16	8.0	20	10.0	36	9.0	
Increase in undesirable effects	16	8.0	12	6.0	28	7.0	
*Attitude in the event of therapeutic failure after herbal treatment*							
Do nothing	45	22.6	51	25.4	96	24.0	*p* = 0.9729
Use fetishists	7	3.5	5	2.5	12	3.0	*r* ^2^ = 0.00010
Use traditional healers	13	6.5	11	5.5	24	6.0	
Change plant	44	22.1	44	21.9	88	22.0	
Use plant mixtures	29	14.6	29	14.4	58	14.5	
Double the dose	31	15.6	32	15.9	63	15.8	
Use of biomedicine	30	15.1	29	14.4	59	14.8	

Abbreviation: Nu, number of informants.

**Table 2:** Medicinal plants used by the people of Mampa village.

**Plant species (family)** ^**MT**^	**Local name (ethnicity)**	**GT**	**Local medical uses ** ^**UP,PF**^	**No. of citations ** **n** = 400** (RFC)**	**No. of uses ** **u** = 32** (UV)**
*Albizia julibrissin* Durazz (Fabaceae)^h^	Musamba mvula (f)	N	Syphilis^k,s^	21 (0.053)	3 (0.0938)
	Mungesha ngesha (d)		Coxarthrosis^k,s^	10 (0.025)	
			Poisoning fish^t,v^	03 (0.008)	

*Anisophyllea pomifera* Engl. & Brehmer⁣^∗^ (Anisophylleaceae)^h^	Lufunga (f)	CA	Urinary infections⁣^∗^^n,r^	46 (0.115)	5 (0.1563)
			**Diabetes** ^m,q^	10 (0.025)	
			**Malaria** ^k,n,q^	05 (0.013)	
			**Cough** ^k,n,k^	04 (0.010)	
			**Hypertension** ^k,n,q^	03 (0.008)	

*Bauhinia variegata* L (Fabaceae)^h^	Ndunda wa bululu (f)	C	Constipation^p,q^	31 (0.078)	1 (0.0313)

*Brachystegia boehmii* Taub (Fabaceae)^h^	Musanga (f)	CA-EA	Constipation^n,s^	46 (0.115)	5 (0.1563)
			**Gonorrhea** ^n,q^	20 (0.050)	
			**Diarrhea** ^l,q^	10 (0.025)	
			Cough^m,r^	15 (0.038)	
			**Wounds** ^m,n,r^	18 (0.045)	

*Buddleja davidii* Franch (Scrophulariaceae)^i^	Sambwe sambwe (f)	B	Hair loss^k,r^	13 (0.033)	2 (0.0625)
			**Wound** ^k,r^	05 (0.013)	

*Butea monosperma* (Lam) Kuntze (Fabaceae)^h^	Mukombo (f)	C	**Snakebite** ^l,t^	12 (0.030)	1 (0.0313)

*Chrysobalanus icaco* L. (Chrysobalanaceae)^i^	Munazi (f)	R	Anemia^k,r^	35 (0.088)	2 (0.0625)
	Kapempe (a)		Intestinal worms^k,r^	27 (0.068)	

*Cratoxylum cochinchinense* (Lour) Blume (Hypericaceae)^h^	Mukute (f)	B	Diarrhea^k,q^	36 (0.068)	2 (0.0625)
	Kafifi (g)		Diarrhea^k,q^	18 (0.045)	

*Crossopteryx febrifuga* (G Don) Benth (Rubiaceae)^h^	Pelapori (f)	P	**Fever** ^l,q^	20 (0.050)	3 (0.0938)
			**Malaria** ^l^	17 (0.043)	
			**Diarrhea** ^l^	19 (0.048)	

*Cupaniopsis anacardioides* (A Rich) Radlk (Sapindaceae)^h^	Mukonda mbazo (f)	D	Tooth decay^l,q^	22 (0.055)	2 (0.0625)
			Decreased libido^n,q^	20 (0.050)	

*Damnacanthus indicus* CF Gaertn (Rubiaceae)^i^	Zanza (f)	C	Swelling pain^k,r^	11 (0.028)	2 (0.0625)
	Sansa (a)		**Cancer** ^k,r^	10 (0.025)	

*Dimocarpus longan* Lour (Sapindaceae)^h^	Mululu (f)	B	Abdominal pain^l,q^	21 (0.053)	1 (0.0313)

*Diospyros virginiana* L. (Ebenaceae)^h^	Bukolongo (f)	K	Syphilis^k,r^	11 (0.028)	1 (0.0313)

*Dombeya rotundifolia* (Hochst) Planch (Malvaceae)^h^	Kiku (f)	F	Vomiting^l,r^	21 (0.053)	1 (0.0313)

*Elaeodendron orientale* Jacq (Celastraceae)^h^	Kakula (f)	M	Anemia^l,r^	12 (0.030)	1 (0.0313)

*Entandrophragma delevoyi* De Wild (Meliaceae)^h^	Kimuti Leza (f)	E	Cough^m,q^	37 (0.093)	4 (0.1250)
			Malaria^m,q^	8 (0.020)	
			Rheumatism^m,q^	6 (0.015)	
			Wound^m,q^	3 (0.008)	

*Flueggea suffruticosa* (Pall) Baill (Phyllanthaceae)^i^	Mupeto wa lupe (f)	A	Swollen fingers^n,t^	11 (0.028)	1 (0.0313)

*Gomphocarpus sinaicus* Boiss [Syn *Asclepias sinaica* (Boiss.) Muschl] (Apocynaceae)^j^	Kapofwe (b)	I	Coxarthrosis^o,q^	21 (0.053)	2 (0.0625)
	Kasobololo (f)		Gastritis^o,q^	19 (0.048)	

*Harungana madagascariensis* Lam ex Poir (Hypericaceae)^h^	Kape (f)	P	**Rheumatism** ^m,r^	8 (0.020)	2 (0.0625)
			Gout^m,r^	15 (0.038)	

*Juglans nigra* L (Juglandaceae)^h^	Mukena mbulo (f)	K	Decreased libido^m,r^	41 (0.103)	2 (0.0625)
			**Diarrhea** ^m,r^	12 (0.030)	

*Kigelia africana* (Lam) Benth (Bignoniaceae)^h^	Muvungu lume (f)	P	Decreased libido^o,r^	21 (0.053)	2 (0.0625)
			Urinary infections⁣^∗^^n,r^	05 (0.013)	

*Landolphia kirkii* Dyer ex Hook.f (Apocynaceae)^i^	Mabungo (a)	E	Fever^n,q^	42 (0.105)	6 (0.1875)
			Pain^n,q^	10 (0.025)	
			Hemorrhoids^†n,q^	18 (0.045)	
			**Malaria** ^n,q^	10 (0.025)	
			Diabetes^n,q^	08 (0.020)	
			Anemia^n,q^	06 (0.015)	

*Parinari curatellifolia* Planch. ex Benth (Chrysobalanaceae)^h^	Mupundu (b)	T	Diarrhea^l,q^	15 (0.038)	1 (0.0313)

*Phoebe lanceolata* (Nees) Nees (Lauraceae)^h^	Kibobo (a)	B	Diarrhea^k,q^	32 (0.080)	2 (0.0625)
	Kakolwa (f)		Wound^l,q^	30 (0.075)	

*Piliostigma reticulatum* (DC.) Hochst (Fabaceae)^h^	Ukifumbe (f)	S	Tooth decay^l,q^	11 (0.028)	2 (0.0625)
			Hemorrhoids^†n,q^	02 (0.005)	

*Piscidia piscipula* (L.) Sarg (Fabaceae)^h^	Mulama (f)	L	Abdominal pain^k,r^	11 (0.028)	1 (0.0313)

*Prunus armeniaca* L. (Rosaceae)^h^	Kasombo (f)	G	Gonorrhea^k,r^	10 (0.025)	1 (0.0313)

*Psidium cattleianum* Sabine (Myrtaceae)^h^	Bupubili (f)	0	Gastritis^m,q^	20 (0.050)	1 (0.0313)

*Pterocarpus angolensis* DC (Fabaceae)^h^	Mukunda mbazu (f)	F	Gout^n,q^	16 (0.040)	2 (0.0625)
			Diabetes mellitus^m,q^	15 (0.038)	

*Pterocarpus brenanii* Barbosa & Torre (Fabaceae)^h^	Kakula (f)	H	Cancer^m,q^	39 (0.098)	4 (0.1250)
			Hepatitis^m,q^	12 (0.030)	
			Malaria^m,q^	06 (0.015)	
			Fever^m,q^	04 (0.010)	

*Pueraria montana* (Lour) Merr (Fabaceae)^j^	Ubupundu (f)	C	Dysentery^k,q^	12 (0.030)	1 (0.0313)

*Quercus suber* L. (Fagaceae)^h^	Ndale (d)	J	Ascites^l,r^	22 (0.055)	2 (0.0625)
	Kilonde (f)		Ascites^m,r^	19 (0.048)	

*Rhododendron simsii* Planch (Ericaceae)^i^	Muda (f)	B	Dental plaque^k,q^	22 (0.055)	2 (0.0625)
			Cough^k,q^	10 (0.025)	

*Ricinus communis* L. (Euphorbiaceae)^h^	Mono (f)	H	Abdominal pain^kr^	13 (0.033)	3 (0.0938)
	Mbalika (e)		Hemorrhoids^n,q^	10 (0.025)	
	Mudia ntondo (c)		Bronchitis^l,q^	12 (0.030)	

*Senegalia polyacantha* (Willd) Seigler & Ebinger (Fabaceae)^h^	Kibombolo (f)	Q	Sexual weakness^m,q^	40 (0.100)	4 (0.1250)
			Diabetes mellitus^m,q^	15 (0.038)	
			Diarrhea^m,q^	20 (0.050)	
			Painful menstruation^m,q^	21 (0.053)	

*Syzygium cumini* (L.) Skeels (Myrtaceae)^h^	Masanfwa (f)	B	Abdominal pain^o,p,u^	31 (0.078)	2 (0.0625)
			Snakebite^n,t^	10 (0.025)	

*Tetradium ruticarpum* (A Juss) TG Hartley (Rutaceae)^h^	Kinsungwa (b)	B	Swelling pain^k,r^	35 (0.088)	2 (0.0625)
	Kinsungu (f)		Headaches^o,s^	15 (0.038)	

*Viburnum odoratissimum* Ker Gawl (Viburnaceae)^i^	Kifubya (f)	B	Abdominal pain^k,q^	21 (0.053)	2 (0.0625)
	Kishimya mulilo (b)		Wounds^l,q^	20 (0.050)	

*Note:* Ethnicity—Bemba: a, Lamba: b, Luba-kas: c, Luba-kat: d, Swahili: e, Tabwa: f, Zela: g. Geographical types (GT)—Asia-temperate: A, Asia-temperate–Asia-tropical: B, Asia-temperate–Asia-tropical–Australia: C, Australia: D, CA-EA: E, CA-SA: F, Central Asia: G, EA: H, NA: I, NA-WE: J, Northern America: K, Northern America–Southern America: L, SA: M, South Asia: N, South America: O, TA: P, TA-SA: Q, WA-CA: R, WA-EA: S, TA-MA: T. Morphological types—tree: h, shrub: i, subshrub: j. Mode of preparation—decoction: q, maceration: r, infusion: s, pillage: t, mastication: u. Used part—root: k, stem root: m, stem bark: l, leaves: n, fruit: o, rhizome: p, seeds: v. Plants used in mixtures (in equal parts)—hemorrhoids: †; urinary infections: ∗relative frequency citation (RFC) = number of people who cited the plant (*ni*) out of the number of people consulted during the survey (*n*). Usual value (UV) = number of uses reported for a plant (*Ui*) out of the total number of uses reported for all plants in the entire study (*u*). Results in bold are expressed as a percentage.

**Table 3:** Extract from the available literature on medicinal uses of plants in the Mampa village.

**Plant**	**Country**	**Previous medicinal uses**	**Source**
*Albizia julibrissin*	China	**Root**: Swelling and pain in the lungs, shin ulcers, wounds, depression	[46]
		**Leaves**: Inflammations	[47]
		**Stem bark**: Ulcers, abscesses, burns, hemorrhoids, and fractures	[48]
	Korea	**Leaves**: Ichthyotoxics	[49]

*Anisophyllea pomifera*	**DRC: Lubumbashi**	**Root**: **Diabetes**, abdominal pain, tooth decay, **malaria**, mental disorders, **hypertension**, **cough**	[35, 36, 50]

*Bauhinia variegata*	India	**Stem bark and flowers**: Obesity, dysentery, hemorrhage	[51]
	India	**Stem bark**: Diarrhea, dysentery, **constipation**, goiter, leprosy, tumor, diabetes, helminth, ulcer, obesity	[52, 53]

*Brachystegia boehmii*	**DRC: Lubumbashi**	**Stem bark**: Dysentery, **diarrhea**, abdominal pain, dysmenorrhea, **gonorrhea**, tuberculosis, typhoid fever	[39]
	Zimbabwe	**Stem bark**: Abdominal pains, antivenom, back pain, cataracts, heart problems, mental problems, sore eyes, toothache, constipation and lumbago in ruminants	[54]
	Zimbabwe	**Leaves**: **Wounds**	[55]
	Mozambique	**Root bark**: Abdominal pains	[56]
	Zambia	**Root bark**: Dizziness and **diarrhea**	[57]

*Buddleja davidii*	China	**Root**: Headache, **wound**	[58]
	Republic of Korea	**Leaf**: Malaria, inflammatory pathologies, **wound**	[59]

*Butea monosperma*	India	**Stem bark**: Helminth, infertility, diabetes, sexual dysfunction	[60]
	India	**Roots**: Impotency, elephantiasis, **snakebite**	[61]

*Chrysobalanus icaco*	Nigeria	**Leaves**: Diabetes, obesity	[62]

*Cratoxylum cochinchinense*	Brazil	**Leaves**: Diabetes	[63]

*Crossopteryx febrifuga*	**DRC: Lubumbashi**	**Stem bark**: Sexual dysfunction, diabetes, **malaria**, hemorrhoids, **diarrhea**, cough, and abdominal pain	[36–38, 41]
	Angola Burkina Faso Congo Ghana Guinea Ivory Coast Mozambique Nigeria Zimbabwe	**Stem bark**: **Malaria**, cough, asthma, pneumonia, tuberculosis, sterility, gastrointestinal complaints, diabetes, wound infections, epilepsy, **fever**	[64]

*Cupaniopsis anacardioides*	Australia	**Fruit, leaves**: Cold, fever, inflammation	[65]

*Damnacanthus indicus*	China	Root: **Cancer**	[66]

*Dimocarpus longan*	China	**Stem bark**: Fatigue	[67]

*Diospyros virginiana*	India	**Root**: Dysentery, diarrhea, fevers, hemorrhoids	[68]

*Dombeya rotundifolia*	RSA	**Stem bark**: Skin infections	[69]

*Elaeodendron orientale*	Madagascar	**Stem bark**: Chest infections, venereal illness, and scorpion fish poisoning	[70]

*Entandrophragma delevoyi*	NR	NR	NR

*Flueggea suffruticosa*	Russia	**Leaves**: Lumbago, rheumatic disease, numbness of the limbs, impotence, infantile paralysis, and indigestion	[71]

*Gomphocarpus sinaicus*	Egypt	**Fruit**: Diarrhea, rhinorrhagia, metrorrhagia	[72]

*Harungana madagascariensis*	Cameroon	**Root bark**: Gonorrhea, leprosy, hemorrhoids, and to facilitate childbirth	[73]
	**DRC: Katanga**	**Root bark**: Diabetes, **rheumatism**, sexual dysfunction, high blood pressure	[36, 41, 43]
*Juglans nigra*	United States	**Root bark**: **Diarrhea**, bilious, cramp colic, and cancers	[74]

*Kigelia africana*	South Africa	**Fruits**: Solar keratosis, malignant melanoma, dysentery, worm infestations, pneumonia, toothache, malaria, diabetes, venereal diseases, convulsions, antidote for snakebite, post parturition hemorrhage, solar keratoses, and skin cancer	[75]
	Botswana	STDs	[75]
	Nigeria	Inflammation diseases	[75]
	**DRC: Lubero**	Epilepsy, hemorrhoid	[76]

*Landolphia kirkii*	**DRC: Lubumbashi**	**Leaves**: **Malaria**, seizures	[34, 37]

*Parinari curatellifolia*	**DRC: Lubumbashi**	**Root bark**: Malaria, **diarrhea**, STDs, hemorrhoids, kunde	[37]

*Phoebe lanceolata*	China	**Root**: **Wound**, cough	[77]

*Piliostigma reticulatum*	Ivory Coast	**Stem bark**: **Diarrhea**	[78]
	Senegal	**Stem bark**: Ulcers, boils, **toothache**, wounds, syphilitic cancer, gingivitis, and **diarrhea**	[79]

*Piscidia piscipula*	Mexico	**Root**: Nerve pain, migraine, insomnia, anxiety, fear, and nervous tension	[80]

*Prunus armeniaca*	Algeria	**Root**: Coughs, bronchitis, asthma, and to soothe inflamed or irritated skin	[81]

*Psidium cattleianum*	Brazil	**Root bark**: Constipation, diarrhea	[82]

*Pterocarpus angolensis*	Angola, India	**Leaf**: Gastrointestinal and urine-genital ailments, fertility problems	[83, 84]
		**Root bark**: Respiratory conditions and skin disorders	[83, 84]
	**DRC: Katanga**	**Root bark**: Malaria, diarrhea, hemorrhoids, wounds, anemia, **diabetes**, tooth decay, hepatitis, fever, otitis	[34, 36–38]

*Pterocarpus brenanii*	NR	NR	NR

*Pueraria montana*		**Root**: Diabetes, gastroenteritis, HTA	[85, 86]

*Quercus suber*	Morocco	**Stem bark**: Diabetes, gastroenteritis, diarrhea, asthma, hemorrhoids, gonorrhea, gastritis, pyrexia	[87]
	Morocco	**Root bark**: Parkinson's disease, hepatoprotective diseases, gastric ulcers	[87]

*Rhododendron simsii*	China	**Root**: Bronchitis, cough, rheumatoid arthritis, pain, and skin ailments	[88]
*Ricinus communis*	India	**Root**: Diabetes, bacterial infections, asthma	[89]
	**DRC: Beni and Lubero**	**Root**: Diabetes	[76]
	India	**Leaf**: Diabetes, helminths, convulsions, ulcers	[89]

*Senegalia polyacantha*	India	**Root bark**: Snakebites, leishmaniasis	[90]
	Benin	**Root bark**: Livestock's diseases	[91]
	Sudan	**Root bark**: STDs, wound, stomach disorders	[92]
	**DRC: Katanga**	**Root bark**: Malaria, **diarrhea**, **diabetes**, female STDs	[34]

*Syzygium cumini*	Philippines, India	**Stem bark**: Dysentery, anemia, gingivitis and mouth ulcerations, diarrhea, diabetes	[93]
	**DRC: Kisangani**	Fruit: Diabetes	[94]
		**Leaf**: Dysentery, stomatitis, vomiting, hemorrhoids	[95]

*Tetradium ruticarpum*	China	**Rhizome**: Gastritis, indigestion, vomiting, gastroduodenal ulcer nausea, neural headache, heart failure, dysmenorrhea	[96, 97]

*Viburnum odoratissimum*	China	**Root**: Rheumatism	[98]

*Note:* Pathologies in bold are those found both in accessible literature and in the Mampa village.

Abbreviations: HTA, high blood pressure; STD, sexually transmitted disease.

**Table 4:** Sociodemographic characteristics of resource persons interviewed.

**Category**	**Household (** **n** = 375**)**		**Herbalists (** **n** = 10**)**		**THs (** **n** = 15**)**		**Total (** **n** = 400**)**		**χ** ^2^ ** test**
	**Nu**	**%**	**Nu**	**%**	**Nu**	**%**	**Nu**	**%**	
*Age*									
20–30	34	**9.1**	0	**0**	1	**6.7**	35	**8.75**	*p* = 0.3762
30–40	155	**41.3**	2	**20**	1	**6.7**	158	**39.5**	*r* ^2^ = 0.0989
40–50	115	**30.7**	4	**40**	3	**20.0**	122	**30.5**	
50–60	59	**15.7**	2	**20**	7	**46.7**	68	**17**	
> 60	12	**3.2**	1	**10**	3	**20.0**	16	**4**	
*Experience (year range)*									
0–5	15	**4.0**	0	**0**	1	**6.7**	16	**4**	*p* = 0.5179
5–10	170	**45.3**	1	**10**	3	**20.0**	174	**43.5**	*r* ^2^ = 0.07289
10–15	168	**44.8**	3	**30**	7	**46.7**	178	**44.5**	
> 15	22	**5.9**	6	**60**	4	**26.7**	32	**8**	
*Main activity*									
Agriculture	30	**8.0**	0	**0**	0	**0.0**	30	**7.5**	*p* = 0.7862
Breeding	155	**41.3**	0	**0**	0	**0.0**	155	**38.75**	*r* ^2^ = 0.00637
Crafts	119	**31.7**	0	**0**	0	**0.0**	119	**29.75**	
Herbalist	0	**0.0**	10	**100**	0	**0.0**	10	**2.5**	
Housework	61	**16.3**	0	**0**	0	**0.0**	61	**15.25**	
THs	0	**0.0**	0	**0**	15	**100.0**	15	**3.75**	
Trade	10	**2.7**	0	**0**	0	**0.0**	10	**2.5**	
*Gender*									
Female	192	**51.2**	6	**60**	3	**20.0**	201	**50.25**	**p** = 0.0412
Male	183	**48.8**	4	**40**	12	**80.0**	199	**49.75**	*r* ^2^ = 0.1282
*Level of education*									
None	61	**16.3**	0	**0**	0	**0.0**	61	**15.25**	*p* = 0.6408
Primary	198	**52.8**	7	**70**	8	**53.3**	213	**53.25**	0.03864
Secondary	115	**30.7**	3	**30**	6	**40.0**	124	**31**	
University	1	**0.3**	0	**0**	1	**6.7**	2	**0.5**	

*Note:* Results in bold are expressed as a percentage.

Abbreviation: Nu, number of informants.

## Data Availability

Since 23 September 2024, this study has been published as a preprint on Research Square (10.21203/rs.3.rs-5116022/v1). The data in support of the conclusions of this study are available on request from the corresponding author.

## Nomenclature

CA: Central Africa
CI: citation index
EA: East Africa
Fr: fruit
FU: form of use
GT: geographical type
Lv: leaves
MA: Madagascar
MCI: medical capability index
MT: morphological type
NA: North Africa
Nc: number of citations
NEA: Northeast Africa
NH: herbarium number
Nt: number of plant
Rb: root bark
Rz: rhizome
SA: Southern Africa
Sb: stem bark
Sd: seed
TA: tropical Africa
TH: type of hemorrhoid
UP: used part
WE: West Africa
WP: whole plant

## References

[1] Payyappallimana U. (2010). The Role of Traditional Medicine in Primary Health Care: An Overview of Perspectives and Challenging. *Yokohama journal of social sciences*.

[2] Kala C. P. (2017). Traditional Health Care Systems and Herbal Medicines. *European Journal of Environment and Public Health*.

[3] Che C. T., George V., Ijinu T. P., Pushpangadan P., Andrae-Marobela K. (2024). Traditional Medicine. *Pharmacogn. (Second Ed. Fundam. Appl. Strateg.)*.

[4] Tesfahuneygn G., Gebreegziabher G. (2019). Medicinal Plants Used in Traditional Medicine by Ethiopians a Review Article. *Journal of Respiratory Medicine and Lung Disease*.

[5] Chaughule R. S., Barve R. S. (2023). Role of Herbal Medicines in the Treatment of Infectious Diseases. *Vegetos*.

[6] Mahomoodally M. F. (2013). Traditional Medicines in Africa: An Appraisal of Ten Potent African Medicinal Plants. *Evidence‐Based Complementary and Alternative Medicine*.

[7] Nasim N., Sandeep I. S., Mohanty S. (2022). Plant-Derived Natural Products for Drug Discovery: Current Approaches and Prospects. *Nucleus*.

[8] Arjona-García C., Blancas J., Beltrán-Rodríguez L. (2021). How Does Urbanization Affect Perceptions and Traditional Knowledge of Medicinal Plants?. *Journal of Ethnobiology and Ethnomedicine*.

[9] Pacios O., Blasco L., Bleriot I. (2020). Strategies to Combat Multidrug-Resistant and Persistent Infectious Diseases. *Antibiotics*.

[10] Terreni M., Taccani M., Pregnolato M. (2021). New Antibiotics for Multidrug-Resistant Bacterial Strains: Latest Research Developments and Future Perspectives. *Molecules*.

[11] Sharma A., Khanna S., Kaur G., Singh I. (2021). Medicinal Plants and Their Components for Wound Healing Applications. *Journal of Pharmaceutical Sciences*.

[12] WHO (2023). *Integrating Traditional and Complementary Medicine Into Health Systems: Social, Economic and Health Considerations*.

[13] Dehelean C. A., Marcovici I., Soica C. (2021). Plant-Derived Anticancer Compounds as New Perspectives in Drug Discovery and Alternative Therapy. *Molecules*.

[14] Pirintsos S., Panagiotopoulos A., Bariotakis M. (2022). From Traditional Ethnopharmacology to Modern Natural Drug Discovery: A Methodology Discussion and Specific Examples. *Molecules*.

[15] Cheung H., Doughty H., Hinsley A. (2021). Understanding Traditional Chinese Medicine to Strengthen Conservation Outcomes. *People and Nature*.

[16] Shi Y., Zhang C., Li X. (2021). Traditional Medicine in India. *Journal of Traditional Chinese Medical Sciences*.

[17] Buso P., Manfredini S., Ahmadi-Ashtiani H. R. (2020). Iranian Medicinal Plants: From Ethnomedicine to Actual Studies. *Medicina*.

[18] Asakitikpi A. (2024). African Indigenous Medicines: Towards a Holistic Healthcare System in Africa. *African Identities*.

[19] Nkwabi S. M., Ngoya A. J. (2022). The Changing Role of Traditional Medicine in the Provision of Health Care in Tanzania from Precolonial to Post-Colonial Period. *Lagos Historical Review*.

[20] Doyle S. (2023). Health in African History. *The History of African Development—An Online Textbook for a New Generation of African Students and Teachers*.

[21] Mutombo C. S., Bakari S. A., Ntabaza V. N. (2022). Perceptions and Use of Traditional African Medicine in Lubumbashi, Haut-Katanga Province (DR Congo): A Cross-Sectional Study. *PLoS One*.

[22] Bashige C. V., Bakari A. S., Ndjolo P. O., Kahumba B. J., Duez P., Lumbu S. J. (2020). Ethnobotanical Study of Plants Used as Antimalarial in Traditional Medicine in Bagira in Eastern RD Congo. *Journal of Pharmacognosy and Phytochemistry*.

[23] Kasali F. M., Kadima J. N., Peter E. L. (2021). Antidiabetic Medicinal Plants Used in Democratic Republic of Congo: A Critical Review of Ethnopharmacology and Bioactivity Data. *Frontiers in Pharmacology*.

[24] Iragi G. K., Imani B., Nfizi I. B. (2021). Ethnomedicinal Study of Plants Used in the Uvira Territory (Democratic Republic of Congo). *Science and Technology*.

[25] Syamasamba M. A., Kapiri M. M., Muhesi K. E., Mbayahi K. E., Mavinga B. M. (2022). Ethnobotanical Study of Plants Used by Traditherapists for the Treatment of Malaria in the City of Butembo, North Kivu, East of the Democratic Republic of Congo, Indones. *Innovative Journal of Applied Science*.

[26] Pathy K. K., Flavien N. B., Honoré B. K., Vanhove W., Patrick V. D. (2021). Ethnobotanical Characterization of Medicinal Plants Used in Kisantu and Mbanza-Ngungu Territories, Kongo-Central Province in DR Congo. *Journal of Ethnobiology and Ethnomedicine*.

[27] Ashande C. M., Ngbolua K. J., Imani B., Rusaati W., Gbolo B. Z. (2023). Ethno-Botanical Survey of Medicinal Plants Species Traditionally Used for the Treatment of Diseases in Kasangulu Territory, DRC, Moroccan. *The Journal of Agricultural Science*.

[28] Bashige C. V., Okusa N. P., Manya M. H., Kasali M. F. (2023). Ethnomedicinal Knowledge of Plants Used in Nonconventional Medicine in the Management of Diabetes Mellitus in Kinshasa (Democratic Republic of the Congo). *Evidence‐Based Complementary and Alternative Medicine*.

[29] Ngunde-Te-Ngunde S., Lengbiye E. M., Ngiala Bongo G. (2020). Ethno-Botanical Survey on Medicinal Plants Traditionally Used to Treat Sickle Cell Anemia in Yakoma Territory (Nord-Ubangi, D. R. Congo). *International Journal of Plant Science and Ecology*.

[30] Ndombe F. M., Ngbolua K., Mawunu M., Da B. Y. (2023). Ethnobotanical, Ecological and Monographic Study of Four Medicinal Plants Traditionally Used in the Treatment of Sterility in Kenge City and Its Surroundings, Democratic Republic of the Congo. *Journal of Applied Biosciences*.

[31] Musuasua M. M., Kabena O. N., Kalanda L. K., Masens D. M. Y., Mpiana P. T. (2021). Floristic and Eco-Morphological Study of Antibacterial Plants in Phytotherapeutic Practice of Kasai Oriental in DR Congo. *Journal of Complementary and Alternative Medical Research*.

[32] Muya K., Tshoto K., Cioci C. C. (2014). Survol ethnobotanique de quelques plantes utilisées contre la schistosomiase urogénitale à Lubumbashi et environs. *Phyothérapie*.

[33] Kalonda E., Mbayo M., Muhume S. (2014). Ethnopharmacological Survey of Plants Used Against Malaria in Lubumbashi City (D.R. Congo). *Journal of Advanced Botany and Zoology*.

[34] Bashige-Chiribagula V., Becker H. S., Youssou Samb B., Jacqueme P. D., Lumbu-Simbi J. B. (2020). Étude ethnobotanique, phytochimique et évaluation de l'activité antiplasmodiale de 13 plantes réputées antipaludéennes dans la commune du Kenya (Lubumbashi, RDC). *Phytothérapie*.

[35] Bashige C. V., Manya M. H., Ntabaza N. V. (2017). Étude ethnobotanique, biologique et chimique de plantes réputées anticariogènes à Lubumbashi–RD Congo. *Phytothérapie*.

[36] Amuri B., Maseho M., Lumbu S., Pierre D., Byanga K. (2018). Ethnobotanical Survey of Herbs Used in the Management of Diabetes Mellitus in Southern Katanga Area/DR Congo. *Pan African Medical Journal*.

[37] Kalonji M., Muya R. K., Mutombo E. K., Cimanga B. C. C., Numbi E. W. I., Kahumba J. B. (2019). Aperçu ethnobotanique de plantes réputées antipaludéennes utilisées dans la ville de Lubumbashi et ses environs , dans le Haut-Katanga en RD Congo. *Ethnopharmacologia*.

[38] Bashige C. V., Alombong A. G., Kamwimba M. A., Bakari A. S., Okusa N. P. (2020). Ethnobotanical Study of Medicinal Plants Used in the Treatment of Sexual Dysfunctions in Traditional Medicine in Kampemba-Lubumbashi, DR Congo. *World Journal of Advanced Research and Reviews*.

[39] Valentin B. C., Pierre K. I., Henry M. M., Mushagalusa F. (2022). Ethnobotanical Study of Plants Used by Traditional Healers in Lubumbashi (Democratic Republic of Congo) in the Management of Typhoid Fever. *Journal of Pharmaceutical Sciences*.

[40] Valentin B. C., Philippe O. N., Henry M. M. (2024). Ethnomedical Knowledge of Plants Used in Nonconventional Medicine for Wound Healing in Lubumbashi, Haut-Katanga Province, DR Congo. *Scientific World Journal*.

[41] François D. O., Eddy N. K., James M. M. (2024). Ethnobotanical Studies of Reputed Aphrodisiac Plants Used in Traditional Medicine in Haut-Katanga in DR of Congo. *International Journal of Innovative Science and Research Technology*.

[42] Bashige C., Bakari A. S., Okusa N. P., Lumbu S. J. (2020). Self-Medication With Antimalarials Drugs in Lubumbashi City (DR Congo). *GSC Biological and Pharmaceutical Sciences*.

[43] Vwakyanakazi M., Petit P. (2004). *Bunganga ya Mici: Guérisseurs et plantes médicinales à Lubumbashi*.

[44] Rea L. M., Parker R. A. (2014). *Designing and Conducting Survey Research: A Comprehensive Guide*.

[45] Leonti M. (2022). The Relevance of Quantitative Ethnobotanical Indices for Ethnopharmacology and Ethnobotany. *Journal of Ethnopharmacology*.

[46] Xian S. H., Oh M., Kwak H. J., Jeong H., Ko H. J., Kim S. H. (2023). Chemical Constituents From the Stem Bark of *Albizia Julibrissin* and Their SREBP-1c Inhibitory Activity. *Journal of Asian Natural Products Research*.

[47] Huang B., Wu Y., Li C., Tang Q., Zhang Y. (2023). Molecular Basis and Mechanism of Action of *Albizia julibrissin* in Depression Treatment and Clinical Application of Its Formulae. *Chinese Herbal Medicines*.

[48] Li W., Yang H. J. (2020). Isolation and Identification of Lignans and Other Phenolic Constituents From the Stem Bark of Albizia julibrissin Durazz and Evaluation of Their Nitric Oxide Inhibitory Activity. *Molecules*.

[49] Cheon I., Song M.-J., Kim H., Lee K. H., Yoo Y. J. (2015). Traditional Knowledge of Plants Used for River Fishing in Local Communities of North Jeolla Province, Korea. *Korean Journal of Community Living Science*.

[50] Bashige-Chiribagula V., Bakari-Amuri S., Mbuyi-Kalonji S., Kahumba-Byanga J., Duez P., Lumbu-Simbi J. B. (2017). Study of the Ethnobotanical, Phytochemical, and Antiplasmodial Activity of Thirteen Medicinal Plants Used Against Malaria in Kenya Commune (Lubumbashi, RDC). *Phytothérapie*.

[51] Sharma K., Kumar V., Kumar S., Sharma R., Mehta C. M. (2021). Bauhinia variegata: A Comprehensive Review on Bioactive Compounds, Health Benefits and Utilization. *Advances in Traditional Medicine*.

[52] Kansal M., Shukla P., Shukla P. (2020). A Boon to Human Health-Bauhinia variegata. *International Journal of Pharmacognosy*.

[53] Nayik G. A., Gull A. (2020). *Antioxidants in Fruits: Properties and Health Benefits*.

[54] Maroyi A. (2023). Medicinal Uses of the Fabaceae Family in Zimbabwe: A Review. *Plants*.

[55] Sibanda S., Shoko R., Chishaya K., Chimwanda P., Nyoni S., Ndlovu J. (2022). Antimicrobial Effect of *Brachystegia boehmii* extracts and Their Green Synthesised Silver Zero-Valent Derivatives on Burn Wound Infectious Bacteria. *All Life*.

[56] Sitoe E., Van Wyk B. (2024). An Inventory and Analysis of the Medicinal Plants of Mozambique. *Journal of Ethnopharmacology*.

[57] Kalirajan A., Kalenshi A., Banda D., Siankuku M. (2021). Phytochemical Screening, Antioxidant and Antibacterial Activity of Leaf Extract of *Morinda citrifolia* L. Against *Escherichia coli* & *Pseudomonas aeruginosa*. *International Journal of Herbal Medicine Journal*.

[58] Chen Y., Montero L., Luo J., Li J., Schmitz O. J. (2020). Application of the New At-Column Dilution (ACD) Modulator for the Two-Dimensional RP×HILIC Analysis of Buddleja davidii. *Analytical and Bioanalytical Chemistry*.

[59] Nguyen A. T., Kim K. Y. (2020). Inhibition of Proinflammatory Cytokines in Cutibacterium acnes-Induced Inflammation in HaCaT Cells by Using Buddleja davidii Aqueous Extract. *International Journal of Inflammation*.

[60] Kumari P., Raina K., Thakur S. (2022). Ethnobotany, Phytochemistry and Pharmacology of Palash (*Butea monosperma* (Lam.) Taub.): A Systematic Review. *Current Pharmacology Reports*.

[61] Pragya L., Sourabh J. (2020). *Butea monosperma* (Lam.) Taub: Review on Its Chemistry, Morphology, Ethnomedical Uses, Phytochemistry and Pharmacological Activities. *Journal of Innovation and Invention in Pharmaceutical Sciences (JIIPS)*.

[62] Onilude H. A., Kazeem M. I., Adu O. B. (2021). *Chrysobalanus icaco*: A Review of Its Phytochemistry and Pharmacology. *Journal of Integrative Medicine*.

[63] Fonseca-Kruel V. S., Neves M. E. R., de Araujo D. S. D., Prance G. T. Ethnobotany of Bajiru (*Chrysobalanus Icaco* L.) In a Coastal Vegetation of Southeastern Brazil. http://www.researchsquare.com/article/9ad3dfa4-280e-46e4-aa31-c41abe8c36d8/v1?utm_source=researcher_app%26utm_medium=referral%26utm_campaign=RESR_MRKT_Researcher_inbound.

[64] Ibekwe N. N. (2022). Crossopteryx febrifuga (Afzel. ex G.Don) Benth: Ethnobotany, Phytochemistry and Pharmacology of an African Tree for Malaria and Beyond. *Tropical Journal of Natural Product Research*.

[65] Ngoc Minh Q. P., Vuong Q. V., Le A. V., Bowyer M. C., Scarlett C. J. (2020). Investigation of the Most Suitable Conditions for Dehydration of Tuckeroo (*Cupaniopsis anacardioides*) Fruits. *Pro*.

[66] Long S., Yuan C., Wang Y., Zhang J., Li G. (2019). Network Pharmacology Analysis of *Damnacanthus indicus C.F.Gaertn* in Gene-Phenotype. *Evidence‐Based Complementary and Alternative Medicine*.

[67] Paul P., Biswas P., Dey D. (2021). Exhaustive Plant Profile of “Dimocarpus longan Lour” With Significant Phytomedicinal Properties: A Literature Based-Review. *Pro*.

[68] Sirisha S. N. V. L., Male A., Sai K. A., Raj I. S., Teja N. B., Ravi G. S. (2018). A Scientific Review on Three Species of Diospyros. *Pharmacognosy Reviews*.

[69] Shikwambana N., Mahlo S. M. (2020). A Survey of Antifungal Activity of Selected South African Plant Species Used for the Treatment of Skin Infections. *Natural Product Communications*.

[70] Jennifer N., Mishra A. P., Nigam M., Devkota H. P., Paudel K. R., Matsabisa M. G. (2022). Bioactive Compounds From the Plants of the *Elaeodendron Genus* and Their Biological Activities—A Review. *Applied Sciences*.

[71] Peng Y., Zeng N., Yao Q. (2023). A Review of the Medicinal Uses, Phytochemistry and Pharmacology of Genus *Flueggea*. *Current Chinese Science*.

[72] Batran M. M., Abdel-Azim N. S., Abdel-Shafeek K. A., Shahat A. E. A., El-Missiry M. M. (2005). Flavonoids of *Gomphocarpus sinaicus* and Evaluation of Some Pharmacological Activities. *Natural Product Sciences*.

[73] Mouthé G., Laure G., Tiani M., Youssouf B., Gbetnkom M. (2020). Phytochemistry and Pharmacology of *Harungana madagascariensis*: Mini Review. *Phytochemistry Letters*.

[74] Khanh-Van H., Anuradha R. (2020). Profiling Anticancer and Antioxidant Activities of Phenolic Compounds Present in Black Walnuts (*Juglans nigra*) Using a High-Throughput Screening Approach. *Molecules*.

[75] Nabatanzi A., Nkadimeng S. M., Lall N., Kabasa J. D., Mcgaw L. J. (2020). Ethnobotany, Phytochemistry and Pharmacological Activity of *Kigelia africana* (Lam.) Benth. (Bignoniaceae). *Plants*.

[76] Kasika E. L., Vasombolwa V. K., Lejoly J. (2016). Popular Medicinal Plants Used by the Bantu People and Pygmies Living in the Administrative Territories of Beni and Lubero (DRC). *Journal of Medicinal Plants Research*.

[77] Semwal D. K., Semwal R. B. (2013). Ethnobotany, Pharmacology and Phytochemistry of the Genus Phoebe (Lauraceae). *Mini-Reviews in Organic Chemistry*.

[78] Guessan B. B. N., Dosso K., Gnangoran B. N. (2015). Antibacterial and Antispasmodic Activities of a Dichloromethane Fraction of an Ethanol Extract of Stem Bark of *Piliostigma reticulatum*. *Journal of Pharmacy & Bioallied Sciences*.

[79] Ibra S., Dieng M., Sarr A., Fall A. D. (2019). Polyphenol Content and Antioxidant Activity of Bark Hydroethanolic Extract of *Piliostigma reticulatum* (DC) Hochst and Its Fractions. *Journal of Medicinal Plants*.

[80] Beytia-Pacheco E. S., Espinoza-Velasco B., Crosby-Galván M. M., Sánchez-Villarreal A., Ramírez-Mella M. (2023). Alimentary and Anti-Methanogenic Potential of Four Species of Tropical Fodder Legumes in Domestic Ruminants. *Agro Product*.

[81] Kitic D., Miladinovic B., Randjelovic M. (2022). Anticancer Potential and Other Pharmacological Properties of *Prunus armeniaca* L.: An Updated Overview. *Plants*.

[82] Faleiro J. H., Gonçalves R. C., Núbia M. (2016). The Chemical Featuring, Toxicity, and Antimicrobial Activity of *Psidium cattleianum* (Myrtaceae) Leaves. *New Journal of Science*.

[83] Santos E. S., Luís Â., Gonçalves J. (2020). *Julbernardia paniculata* and *Pterocarpus angolensis*: From Ethnobotanical Surveys to Phytochemical Characterization and Bioactivities Evaluation. *Molecules*.

[84] Chowdhury S. R., Haldar S., Bhar R. (2022). Pterocarpus angolensis: Botanical, Chemical and Pharmacological Review of an Endangered Medicinal Plant of India. *Journal of Experimental Biology and Agricultural Sciences*.

[85] Cui T., Tang S., Liu C. (2018). Three New Isoflavones From the *Pueraria montana var. lobata* (Willd.) and Their Bioactivities. *Natural Product Research*.

[86] Zhang G., Liu J., Gao M. (2020). Tracing the Edible and Medicinal Plant Pueraria montana and Its Products in the Marketplace Yields Subspecies Level Distinction Using DNA Barcoding and DNA Metabarcoding. *Frontiers in Pharmacology*.

[87] Taib M., Rezzak Y., Bouyazza L., Lyoussi B. (2020). Medicinal Uses, Phytochemistry, and Pharmacological Activities of Quercus Species. *Evidence‐Based Complementary and Alternative Medicine*.

[88] Wang F., Sun W., Lan Z. (2023). Chemical Constituents and Hepatoprotective Properties of *Rhododendron simsii* Planch Extract in Con A-Induced Autoimmune Hepatitis. *Arabian Journal of Chemistry*.

[89] Chouhan H. S., Swarnakar G., Jogpal B. (2021). Medicinal Properties of *Ricinus communis*: A Review. *International Journal of Pharmaceutical Sciences and Research*.

[90] Salbei C., Ngeiywa M., Makwali J. (2020). Efficacy of Crude Bark Extract of Acacia polyacantha Against Leishmania donovani in Mice. *International Journal of Sciences: Basic and Applied Research (IJSBAR)*.

[91] Alain K. Y., Dossa A., Pascal C. (2015). Chemical Characterization and Biological Activities of Extracts From Two Plants (*Cissus quadrangularis* and *Acacia polyacantha*) Used in Veterinary Medicine in Benin. *Journal of Pharmacognosy and Phytochemistry*.

[92] Daffalla H. M., Sir K., Ali E. (2019). Larvicidal and Antibacterial Activities of Methanol Extract of *Acacia polyacantha* Willd. *Journal of Advanced Research in Pharmaceutical Sciences & Pharmacology Interventions*.

[93] Kumar S., Singh B. (2021). *Syzygium cumini* (Jamun) Its Medicinal Uses. *International Journal of Pharmacognosy*.

[94] Katemo M., Mpiana P. T., Mbala B. M. (2012). Ethnopharmacological Survey of Plants Used Against Diabetes in Kisangani City (DR Congo). *Journal of Ethnopharmacology*.

[95] Nadeem A., Mohammad N., Husain Mohammad K. M., Husain K. M. (2019). Medicinal Potential of Jamun (*Syzygium cumini Linn*): A Review. *Journal of Drug Delivery & Therapeutics*.

[96] Li M., Wang C. (2020). Traditional Uses, Phytochemistry, Pharmacology, Pharmacokinetics and Toxicology of the Fruit of *Tetradium ruticarpum*: A Review. *Journal of Ethnopharmacology*.

[97] Shan Q., Sang X., Hui H. (2020). Processing and Polyherbal Formulation of *Tetradium ruticarpum* (A. Juss.) Hartley: Phytochemistry, Pharmacokinetics, and Toxicity. *Frontiers in Pharmacology*.

[98] Zhang Y., Zhou W., Song X. (2020). Neuroprotective Terpenoids From the Leaves of *Viburnum odoratissimum*. *Natural Product Research*.

[99] Mohammed M., Akeel A., Marzuq W. (2018). Herbal Medicines: Saudi Population Knowledge, Attitude, and Practice at a Glance. *Journal of Family Medicine and Primary Care*.

[100] Welz A. N., Emberger-klein A., Menrad K. (2018). Why People Use Herbal Medicine: Insights From a Focus-Group Study in Germany. *BMC Complementary and Alternative Medicine*.

[101] Waweru D., Otieno M., Gitahi S. (2020). Traditional Medicine in Kenya: Past and Current Status, Challenges, and the Way Forward. *Scientific African*.

[102] Anywar G., Kakudidi E., Byamukama R. (2021). A Review of the Toxicity and Phytochemistry of Medicinal Plant Species Used by Herbalists in Treating People Living With HIV/AIDS in Uganda. *Frontiers in Pharmacology*.

[103] Rondilla N. A. O., Rocha I. C. N., Roque S. J. R. (2021). Folk Medicine in the Philippines: A Phenomenological Study of Health-Seeking Individuals. *International Journal of Medical Students*.

[104] Marques B., Freeman C., Carter L. (2022). Adapting Traditional Healing Values and Beliefs Into Therapeutic Cultural Environments for Health and Well-Being. *International Journal of Environmental Research and Public Health*.

[105] Suharti B., Kartika T. (2021). Culture and Social: Herbal Medicine as Health Communication to Build Urban Community Empowerment. *Jurnal Studi Komunikasi*.

[106] Nkengurutse J., Mansouri F., Bekkouch O. (2019). Chemical Composition and Oral Toxicity Assessment of *Anisophyllea boehmii* Kernel Oil: Potential Source of New Edible Oil With High Tocopherol Content. *Food Chemistry*.

[107] Tan M., Otake Y., Tamming T., Akuredusenge V., Uwinama B., Hagenimana F. (2021). Local Experience of Using Traditional Medicine in Northern Rwanda: A Qualitative Study. *BMC Complementary Medicine and Therapies*.

[108] Bazie G. W., Adimassie M. T. (2017). Modern Health Services Utilization and Associated Factors in North East Ethiopia. *PLoS One*.

[109] Chali B. U., Hasho A., Koricha N. B. (2021). Preference and Practice of Traditional Medicine and Associated Factors in Jimma Town, Southwest Ethiopia. *Evidence‐Based Complementary and Alternative Medicine*.

[110] Aina O., Gautam L., Simkhada P., Hall S. (2020). Prevalence, Determinants and Knowledge About Herbal Medicine and Non-Hospital Utilisation in Southwest Nigeria: A Cross-Sectional Study. *BMJ Open*.

[111] Périne M. N. A., Ache S. B., Mireille A., Nga N., Serge A. A., Joseph N. (2021). Knowledge, Attitudes, Practices on Homologation of Improved Traditional Medicines Among Practitioners in Mfoundi Division of Cameroon. *International Journal of Pharmaceutical and Phytopharmacological Research (eIJPPR)*.

[112] Nsagha D. S., Ayima C. W., Nana-Njamen T., Assob J. C. N. (2020). The Role of Traditional, Complementary/Alternative Medicine in Primary Healthcare, Adjunct to Universal Health Coverage in Cameroon: A Review of the Literature. *American Journal of Epidemiology*.

[113] Nang C., Namutambo Y., Mufwambi W. (2022). Prevalence and Patterns of Herbal Medicine Use Among Type 2 Diabetes Mellitus Patients at the University Teaching Hospitals in Lusaka. *Journal of Biomedical Research & Environmental Sciences*.

[114] Tosam M. J. (2019). Human Nature, Disease Diagnosis and Health in Traditional African Medicine. *Polylog*.

[115] Anywar G., Kakudidi E., Byamukama R. (2021). A Review of the Toxicity and Phytochemistry of Medicinal Plant Species Used by Herbalists in Treating People Living With HIV/AIDS in Uganda. *Frontiers in Pharmacology*.

[116] Ijaz N., Hunter J., Grant S., Templeman K. (2024). Protocol for a Scoping Review of Traditional Medicine Research Methods, Methodologies, Frameworks and Strategies. *Frontiers in Medicine*.

[117] Luo L., Wang B., Jiang J. (2021). Heavy Metal Contaminations in Herbal Medicines: Determination, Comprehensive Risk Assessments, and Solutions. *Frontiers in Pharmacology*.

[118] Okaiyeto K., Oguntibeju O. O. (2021). African Herbal Medicines: Adverse Effects and Cytotoxic Potentials With Different Therapeutic Applications. *International Journal of Environmental Research and Public Health*.

[119] Mbayo K. M., Kalonda M. E., Tshisand T. P. (2016). Contribution to Ethnobotanical Knowledge of Some Euphorbiaceae Used in Traditional Medicine in Lubumbashi and Its Surroundings (DRC). *Journal of Advanced Botany and Zoology*.

[120] Okombe V., Simbi J. L., Stévigny C., Vandenput S., Pongombo C., Duez P. (2014). Traditional Plant-Based Remedies to Control Gastrointestinal Disorders in Livestock in the Regions of Kamina and Kaniama (Katanga Province, Democratic Republic of Congo). *Journal of Ethnopharmacology*.

[121] Bashige C. V., Bakari S., Amuri K. S., Mbuyi B. J., Kahumba P., Duez S. J. L. (2020). Étude ethnobotanique, phytochimique et évaluation de l’activité antiplasmodiale de 13 plantes réputées antipaludéennes dans la commune du Kenya (Lubumbashi, RDC). *Phytothérapie*.

[122] Kacholi D. S., Mvungi Amir H. (2022). Herbal Remedies Used by Traditional Healers to Treat Haemorrhoids in Tabora Region, Tanzania. *Pharmaceutical Biology*.

[123] Novotna B., Polesny Z., Pinto-Basto M. F. (2020). Medicinal Plants Used by ‘Root Doctors’, Local Traditional Healers in Bié Province, Angola. *Journal of Ethnopharmacology*.

[124] Nyirenda J., Chipuwa M. (2024). An Ethnobotanical Study of Herbs and Medicinal Plants Used in Western, Copperbelt, Central and Northern Provinces of Zambia. *Phytomedicine Plus*.

[125] Chikowe I., Mnyenyembe M., Jere S., Mtewa A., Mponda J., Lampiao F. (2020). An Ethnomedicinal Survey of Indigenous Knowledge on Medicinal Plants in the Traditional Authority Chikowi in Zomba, Malawi. *Current Traditional Medicine*.

[126] Shopo B., Mapaya R., Maroyi A. (2022). Ethnobotanical Study of Medicinal Plants Traditionally Used in Gokwe South District, Zimbabwe. *South African Journal of Botany*.

[127] Bashige V. C., Philippe O. N., Henry M. M., Salvius B. A., Suzanne M. K., Kasali M. (2024). Ethnomedical Knowledge of Plants Used in Nonconventional Medicine for Wound Healing in Lubumbashi, Haut-Katanga Province, DR Congo. *Scientific World Journal*.

[128] Van Wyk B. E. (2020). A Family-Level Floristic Inventory and Analysis of Medicinal Plants Used in Traditional African Medicine. *Journal of Ethnopharmacology*.

[129] Valentin B. C., Philippe O. N., Melman M., Henry M. M., Salvius B. A., Baptiste L. S. J. (2024). Ethnomedical Knowledge of Plants Used in Alternative Medicine to Treat Hemorrhoidal Diseases in Lubumbashi, Haut-Katanga Province, Southern Democratic Republic of Congo. *BMC Complementary Medicine and Therapies*.

[130] Abbafati C., Abbas K. M., Abbasi-Kangevari M. (2020). Global Burden of 369 Diseases and Injuries in 204 Countries and Territories, 1990–2019: A Systematic Analysis for the Global Burden of Disease Study 2019. *Lancet*.

[131] Ohemu T. L., Shalkur D., Ohemu B. O., Daniel P. (2021). Knowledge, Attitude and Practice of Traditional Medicine Among People of Jos South Local Government Area of Plateau State, Nigeria. *Journal of Pharmacy & Bioresources*.

[132] Care P., Gizaw Z., Astale T., Kassie G. M. (2022). What Improves Access to Primary Healthcare Services in Rural Communities ? A Systematic Review. *BMC Primary Care*.

[133] Cordero C. S., Meve U., Jonathan G., Alejandro D. (2023). Ethnobotany and Diversity of Medicinal Plants Used Among Rural Communities in Mina, Iloilo, Philippines: A Quantitative Study. *Journal of Asia-Pacific Biodiversity*.

[134] Maloba M. J., Muyumba N. W., Kasanya K. J. (2020). Ethnobotanical Survey and Preliminary Chemical Screening of 14 Plants Reputed Antigastritis in Lubumbashi and Surroundings (DR Congo). *International European Extended Enablement in Science, Engineering & Management*.

[135] Scott G. J. (2021). A Review of Root, Tuber and Banana Crops in Developing Countries: Past, Present and Future. *International Journal of Food Science and Technology*.

[136] Hussein R. A., El-Anssary A. A. (2021). Plants Secondary Metabolites: The Key Drivers of the Pharmacological Actions of Medicinal Plants. *Herbal Medicine*.

[137] Rasul M. G. (2019). Extraction, Isolation and Characterization of Natural Products From Medicinal Plants. *International Journal of Basic Sciences and Applied Computing (IJBSAC)*.

[138] El Maaiden E., Bouzroud S., Nasser B. (2022). A Comparative Study Between Conventional and Advanced Extraction Techniques: Pharmaceutical and Cosmetic Properties of Plant Extracts. *Molecules*.

[139] Bitwell C., Sen Indra S., Luke C., Kakoma M. K. (2023). A Review of Modern and Conventional Extraction Techniques and Their Applications for Extracting Phytochemicals From Plants. *Scientific African*.

[140] Hakizimana P., Masharabu T., Bangirinama F., Habonimana B., Bogaert J. (2011). Analyse du rôle de la biodiversité végétale des forêts de Kigwena et de Rumonge au Burundi. *Tropicultura*.

[141] Mpasiwakomu R. (2021). *The Diversity and Utilization of Wild Medicinal Plant Species Found in the Miombo Woodlands of Uvinza, Tanzania*.

[142] Moura I., Duvane J. A., Silva M. J., Ribeiro N., Isabel A. (2018). Woody Species From the Mozambican Miombo Woodlands: A Review on Their Ethnomedicinal Uses and Pharmacological Potential. *Journal of Medicinal Plants Research*.

[143] Bruschi P., Mancini M., Mattioli E., Morganti M., Signorini M. A. (2014). Traditional Uses of Plants in a Rural Community of Mozambique and Possible Links With Miombo Degradation and Harvesting Sustainability. *Journal of Ethnobiology and Ethnomedicine*.

[144] Maroyi A. (2013). Traditional Use of Medicinal Plants in South-Central Zimbabwe: Review and Perspectives. *Journal of Ethnobiology and Ethnomedicine*.

[145] Chirisa S., Mukanganyama E. (2016). Evaluation of In Vitro Anti-Inflammatory and Antioxidant Activity of Selected Zimbabwean Plant Extracts. *Journal of Herbs Spices & Medicinal Plants*.

[146] Duvane J. A., Jorge T. F., Maquia I., Ribeiro N., Ribeiro-Barros A. I. F., António C. (2017). Characterization of the Primary Metabolome of Brachystegia boehmii and Colophospermum mopane Under Different Fire Regimes in Miombo and Mopane African Woodlands. *Frontiers in Plant Science*.

[147] Mbuyi K. S., Kalunga M. R., Kalonda M. (2019). Aperçu ethnobotanique de plantes réputées antipaludéennes utilisées dans la ville de Lubumbashi et ses environs, dans le Haut-Katanga en RD Congo. *Ethnopharmacologia*.

[148] Corrigan B. M., Van Wyk C. J., Geldenhuys B. E., Jardine J. M. (2011). Ethnobotanical Plant Uses in the KwaNibela Peninsula, St Lucia, South Africa. *South African Journal of Botany*.

[149] Zwane N. S., De Wet H., Van Vuuren S. F. (2024). Blood Purification Practices: Some Ethnopharmacological Insight From a Rural Community in KwaZulu-Natal, South Africa. *Journal of Ethnopharmacology*.

[150] Pakia M., Cooke J. A., van Staden J. (2003). The Ethnobotany of the Midzichenda Tribes of the Coastal Forest Areas in Kenya: 2. Medicinal Plant Uses. *South African Journal of Botany*.

[151] Mutie F. M., Mbuni Y. M., Rono P. C. (2023). Important Medicinal and Food Taxa (Orders and Families) in Kenya, Based on Three Quantitative Approaches. *Plants*.

[152] Mboni H. M., Keymeulen F., Ngezahayo J. (2020). Antimalarial Herbal Remedies of Bukavu and Uvira Areas in DR Congo: An Ethnobotanical Survey. *Journal of Ethnopharmacology*.

[153] Baumgärtel T., Lautenschläger T. (2023). The Genus *Landolphia* P.Beauv. (Apocynaceae): A Comprehensive Review on Its Ethnobotanical Utilizations, Pharmacology and Nutritional Potential. *Journal of Ethnopharmacology*.

[154] Tshikalange T. E., Modishane D. C., Tabit F. T. (2017). Antimicrobial, Antioxidant, and Cytotoxicity Properties of Selected Wild Edible Fruits of Traditional Medicinal Plants. *Journal of Herbs Spices & Medicinal Plants*.

[155] Matemu A. O., Adeyemi D., Nyoni H. (2017). Fatty Acid Composition of Dried Fruits of *Sclerocarya birrea*, *Diospyros blancoi* and *Landolphia kirkii*. *International Journal of Environmental Research and Public Health*.

[156] Mwamatope B., Chikowe I., Tembo D. T., Kamanula J. F., Masumbu F. F. F., Kumwenda F. D. (2023). Phytochemical Composition and Antioxidant Activity of Edible Wild Fruits From Malawi. *BioMed Research International*.

[157] Mulholland D. A., Schwikkard S. L., Sandor P., Nuzillard J. M. (2000). Delevoyin C, a Tetranortriterpenoid From *Entandrophragma delevoyi*. *Phytochemistry*.

[158] Happi G. M., Ngadjui B. T., Green I. R., Kouam S. F. (2018). Phytochemistry and Pharmacology of the Genus Entandrophragma Over the 50 Years From 1967 to 2018: A ‘Golden’ Overview. *The Journal of Pharmacy and Pharmacology*.

[159] Saslis-Lagoudakis C. H., Klitgaard B. B., Forest F. (2011). The Use of Phylogeny to Interpret Cross-Cultural Patterns in Plant Use and Guide Medicinal Plant Discovery: An Example From Pterocarpus (Leguminosae). *PLoS One*.

[160] Islam M. R., Akbar S., Akter B., Nahar A., Proma N. M., Hossain M. K. (2023). Phytochemical Screening and Evaluation of In-Vivo Analgesic Activity of Pterocarpus indicus Leaf. *Chittagong University Journal of Biological Sciences*.

